# Attention-Based Variational Autoencoder Models for Human–Human Interaction Recognition via Generation

**DOI:** 10.3390/s24123922

**Published:** 2024-06-17

**Authors:** Bonny Banerjee, Murchana Baruah

**Affiliations:** Institute for Intelligent Systems, and Department of Electrical & Computer Engineering, University of Memphis, Memphis, TN 38152, USA; murchanabaruah@gmail.com

**Keywords:** embodied AI agent, intent prediction, human–human interaction recognition, human–human interaction generation, attention, perception, proprioception, multimodal, variational autoencoder, recurrent neural network (RNN), long-short term memory (LSTM)

## Abstract

The remarkable human ability to predict others’ intent during physical interactions develops at a very early age and is crucial for development. Intent prediction, defined as the simultaneous recognition and generation of human–human interactions, has many applications such as in assistive robotics, human–robot interaction, video and robotic surveillance, and autonomous driving. However, models for solving the problem are scarce. This paper proposes two attention-based agent models to predict the intent of interacting 3D skeletons by sampling them via a sequence of glimpses. The novelty of these agent models is that they are inherently multimodal, consisting of perceptual and proprioceptive pathways. The action (attention) is driven by the agent’s generation error, and not by reinforcement. At each sampling instant, the agent completes the partially observed skeletal motion and infers the interaction class. It learns where and what to sample by minimizing the generation and classification errors. Extensive evaluation of our models is carried out on benchmark datasets and in comparison to a state-of-the-art model for intent prediction, which reveals that classification and generation accuracies of one of the proposed models are comparable to those of the state of the art even though our model contains fewer trainable parameters. The insights gained from our model designs can inform the development of efficient agents, the future of artificial intelligence (AI).

## 1. Introduction

Humans possess a remarkable ability to predict the intentions of others during physical interactions, a skill that is crucial for seamless social interactions, collaborative tasks, and competitive scenarios [1,2,3,4]. The ability to perceive others as intentional agents is innate and crucial to development [5]. Humans begin to understand others’ intentions during physical interactions within the first year of life. Infants start to attribute intentions to others’ actions as they develop their motor skills and engage in social interactions. By around five months of age, infants begin to produce smooth object-directed reaches, which is a milestone in their ability to produce coordinated goal-directed actions [6]. This development in their actions could provide information to structure infants’ perception of others’ actions, suggesting that as infants become more capable of intentional actions such as reaching or tool use, they may also start to understand the intentions behind others’ actions [6].

In artificial intelligence (AI) and related areas, human intent prediction has been extensively studied in the context of different applications such as assistive robotics (e.g., [7]), human-robot interaction (e.g., [8]), video and robotic surveillance (e.g., [9]), and autonomous driving (e.g., [10]). Following [11], we define *“intent prediction” as the problem of simultaneously inferring the action/interaction class and generating the involved persons’ future body motions*. Models that perform both generation and recognition of human-human interactions are scarce.

This paper proposes two attention-based agent models that sample 3D skeleton(s) via a sequence of glimpses for predicting the intent of the skeleton(s). The models implement a perception-action loop to optimize an objective function. At each sampling instant, the models predict the interaction class and complete the partially observed skeletal motion pattern. *The action (attention) is modeled as proprioception in a multimodal setting and is guided by perceptual prediction error, not by reinforcement*. This kind of embodied agent model was first introduced in [12], and has since been used for handwriting generation from images and videos [13], handwriting recognition [14], human interaction generation [15], human interaction recognition [11], and speech emotion recognition [16]. As in [11], at each sampling instant, our models simultaneously predict the interaction class and the motion of both 3D skeletons. The models are used in both *first-person* (FP) and *third-person* (TP) environments. Unlike large AI models, the proposed models actively and selectively sample their environment, which allows them to be efficient in terms of model size (number of trainable parameters), data size (number of skeleton joints sampled at each glimpse on average), and training time. On comparing the proposed models (say, M2 and M3) with that in [11] (say, M1), our findings are as follows:

1. The efficiency, and generation and classification accuracy on benchmark datasets of the three models (M1, M2, M3) are analyzed in both FP and TP environments. M1 yields the highest classification accuracy, followed closely by M2. In each environment, the accuracies are correlated with the number of trainable parameters. No model is the clear winner for generation accuracy.
2. Three action selection methods (where to attend to) are analyzed for each of M1, M2, M3. Classification accuracy is comparable when sampling locations are determined from prediction error (without any weighting) or from learned weights (without involving prediction error); however, the latter is less efficient in terms of model size.

The rest of this paper is organized as follows. The next section presents a review of the literature on related work. The proposed agent models are described in Section 3 and evaluated on benchmark datasets in Section 4. The paper concludes in Section 6. Objective function derivations are included in the Appendix A.

## 2. Related Work

While a number of models have been reported for intent prediction from body motions and/or eye gaze (see [17,18] for related reviews), few of them perform action classification and generation simultaneously. A large volume of work has been reported on generating actions using only one 3D skeleton (e.g., [15,19,20,21]) or on generating human motion in crowded scenes (e.g., [22,23,24,25,26,27]). Comparatively, much less has been reported on generating interaction of two persons using 3D skeletal data (e.g., [28,29,30]).

The models in [28,30] generate the 3D pose of one of the skeletons upon observing the motions of the other. Given a sequence of 3D skeletal interactions of two persons, the model in [29] generates their 3D skeletal interaction data for future time-steps. Some of these models use attention. For example, temporal attention is used in [21,25], an attention mechanism that weighs different modalities is used in [22,23], and spatiotemporal attention is used in [24].

There is also a large volume of work on two-person interaction classification from videos (e.g., [31]) and skeletal data (e.g., [32,33,34,35,36,37,38,39]). Some of these models incorporate temporal [31,37], spatial and temporal [34], or multilayer feature [35] attention mechanisms.

Models for two-person interaction generation (e.g., [11,15,29,40]), reaction generation (e.g., [28,30,41,42]), and two-person interaction recognition (e.g., [11,32,34,35,37,38,39]) using 3D skeletal data have been widely reported in the artificial intelligence (AI) and machine learning (ML) literature. Interaction generation is more challenging than reaction generation as the former requires generating the interaction sequence of both skeletons, while the latter requires generating the reaction sequence of one skeleton given the action sequence of the other.

As noted in [11], the environment in these works is viewed from one of two perspectives: first person (FP), where one of the interacting persons is the observer while the other constitutes his environment (e.g., [11,28,30,42]), or third person (TP), where a person, such as an audience, is the observer and the two interacting persons constitute the observer’s environment (e.g., [11,29]).

Very few end-to-end AI/ML models perform both generation and recognition. In a model, generation and recognition can be performed either separately, such as in [41], or simultaneously, such as in [11,42] and our current work. In [11], both interacting skeletons in both FP and TP are generated by utilizing a variational recurrent neural network (RNN)-based model. In [42], only the reacting skeleton in FP is generated using a generative adversarial network. To the best of our knowledge, the model reported in [11] is the only one that performs simultaneous generation and recognition of two-person interactions.

Some of these models are attention-based. They utilize different attention mechanisms, such as temporal (e.g., [21,25,31,37,42]), spatiotemporal (e.g., [11,24,34]), multimodal (e.g., [22,23]), or multilayer (e.g., [35]). In most models, attention is implemented by strategically introducing additional learnable parameters. For example, a transformer-based attention mechanism is used in [41], and a sequence-to-sequence long short-term memory (LSTM)-based attention layer is used in [42], both of which introduce additional attention parameters learned during training. As a consequence, the model size may increase exorbitantly to the extent that the execution of its software code for learning and inference requires specialized hardware resources, as in the case of many transformer-based large language models. In [11] as well as in this paper, attention is computed directly from the generation error, which is why generation is necessary. Learnable attention parameters may or may not be used in the models in [11] and in this paper. We show that these models yield state-of-the-art recognition accuracy while being efficient, and learnable attention parameters to weigh the generation error do *not* increase the accuracy on benchmark datasets.

## 3. Models and Methods

### 3.1. Preliminaries

This section defines a few concepts that are well established in the field and form the basis of this paper, so that there is no ambiguity in the meaning of these terms.

**Agent:** An agent is anything that can be viewed as perceiving its environment through sensors and acting upon that environment through actuators [43].

**Perception** is the mechanism that allows an agent to interpret sensory signals from the external environment [44].

**Proprioception** is perception where the environment is the agent’s own body [12]. Proprioception allows an agent to internally perceive the location and movement of parts of its body [44].

**Generative model:** A generative model, $p_{model}$, maximizes the log-likelihood L(x;θ) of the data, where θ is a set of parameters and *x* is a set of data points [45].

**Evidence lower bound (ELBO):** If *z* is a latent continuous random variable generating the data *x*, computing log-likelihood requires computing the integral of the marginal likelihood, $\int p_{model}\left(x,z\right)dz$, which is intractable [46]. Variational inference involves optimization of an approximation of the intractable posterior by defining an evidence lower bound (ELBO) on the log-likelihood, $L\left(x;\theta \right)\leq \log p_{model}\left(x;\theta \right)$.

**Variational autoencoder (VAE)** is a deep generative model that assumes the data consist of independent and identically distributed samples, and the prior, $p_{\theta }\left(z\right)$, is an isotropic Gaussian. VAE maximizes the ELBO given by [46]:

(1) $$L\left(x;\theta \right)\leq E_{q_{\phi }\left(z|x\right)}\left[\log\;p_{\theta }\left(x|z\right)\right]-D_{K}L\left(q_{\phi }\left(z|x\right),p_{\theta }\left(z\right)\right)$$

where $q_{\phi }\left(z|x\right)$ is a recognition model, $p_{\theta }\left(x|z\right)$ is a generative model, E denotes expectation, and $D_{K}L$ denotes Kullback–Leibler divergence.

**Saliency** is a property of each location in a predictive agent’s environment. The attention mechanism is a function of the agent’s prediction error [47,48].

### 3.2. Problem Statement

Let $X=\{X^{\left(1\right)},X^{\left(2\right)},\ldots ,X^{\left(n\right)}\}$ be a set of observable variables representing an environment in *n* modalities (or signal types or sources). The variable representing the *i*-th modality is a sequence: $X^{\left(i\right)}=\langle X_{1}^{\left(i\right)},X_{2}^{\left(i\right)},\ldots ,X_{T}^{\left(i\right)}\rangle$, where *T* is the sequence length. Let $x_{\leq t}=\left\{x^{\left(1\right)},x^{\left(2\right)},\ldots ,x^{\left(n\right)}\right\}$ be a partial observation of X such that $x^{\left(i\right)}=\langle x_{1}^{\left(i\right)},\ldots ,x_{t}^{\left(i\right)}\rangle$, 1≤t≤T. Let y be a variable representing the class labels. Following [11], we define the problem of *pattern completion and classification* as generating X and y as accurately as possible from the partial observation $x_{\leq t}$. Given $x_{\leq t}$ and a generative model $p_{\theta }$ with parameters θ, at any time *t*, the objective is to maximize the joint likelihood of X and y, i.e., $\arg\max_{\theta }p_{\theta }\left(X,y|x_{\leq t}\right)$.

### 3.3. Models

**Figure 1:**
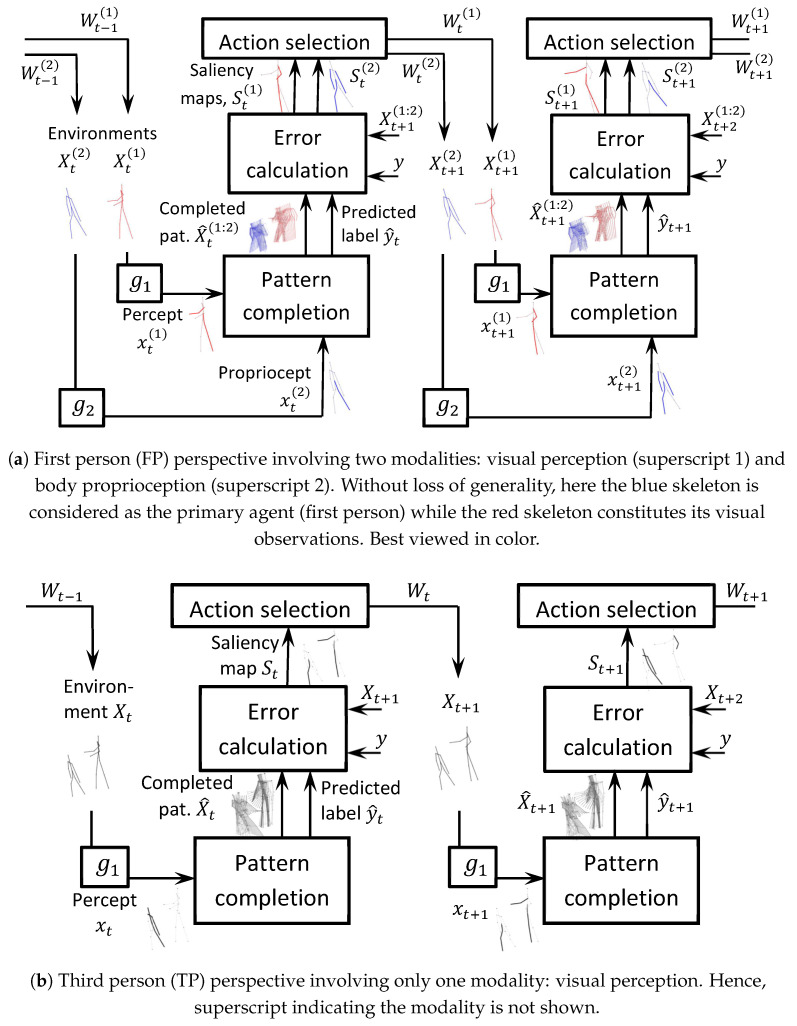
Block diagrams of the proposed attention-based agent applied to two-person interaction generation and classification. In the benchmark skeleton datasets, there is no information regarding the appearance of joints (shape, color, texture) but only their location. The appearance constitutes visual perception (‘what’) while location constitutes visual proprioception (‘where’). The mathematical symbols used in the diagrams are defined in Section 3.

We present two models (M2, M3) for solving this problem and closely compare them with the model (M1) in [11]. See Figure 1 for block diagrams of the agent within which these models reside.

**Model M1.** [11] The completed pattern and class label are generated from the latent variable $z_{\leq t}$. Mathematically,

(2) $$\arg\;\max_{\theta }\int log\left(p_{\theta }\left(X|x_{<t},z_{\leq t}\right)p_{\theta }\left(z_{\leq t}\right)\right)dz+\arg\;\max_{\theta }\int log\left(p_{\theta }\left(y|x_{<t},z_{\leq t}\right)p_{\theta }\left(z_{\leq t}\right)\right)dz$$

**Figure 2:**
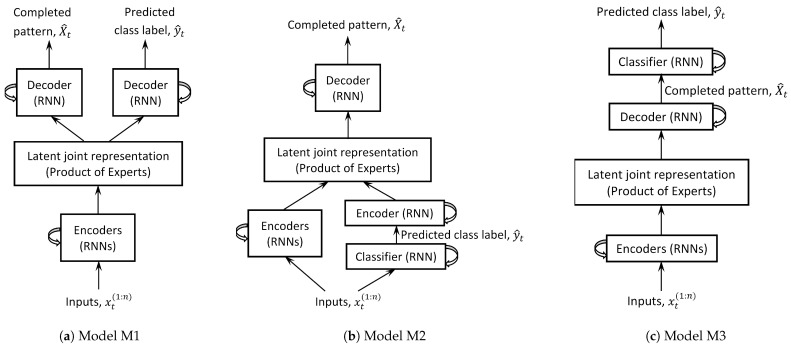
Implementation of the “pattern completion” block (ref. Figure 1) for the three models considered in this paper are shown. The number of encoders (RNNs) is equal to the number of input modalities (*n*); one encoder for each input modality. Inputs, completed pattern, and predicted class label are the same for the three models, shown for time step *t*. Model M1 was proposed in [11] while M2 and M3 are proposed in this paper.

The model is trained end-to-end. See Figure 2a. The pseudocodes, borrowed from [11], are shown in Algorithms 1 and 2.
**Algorithm 1** Learning the proposed network  1:Initialize parameters of the generative model θ, recognition model ϕ, sequence length *T*.  2:Initialize optimizer parameters: $\beta_{1}=0.9$, $\beta_{2}=0.99$, η=0.001, $\epsilon =10^{-10}$.  3:Initialize $W_{0}$ values as 1 and $x_{1}^{\left(1:2\right)}\leftarrow F\left(X_{1}^{\left(1:2\right)},W_{0}^{\left(1:2\right)}\right)$, where $W_{0}^{\left(1:2\right)}$ are the weights for the initial sampling (ref. experimental setup in Section 4.2) and the function *F* generates a sample $x^{\left(i\right)}$ from the environment $X^{\left(i\right)}$ after assigning weights $W_{0}^{\left(i\right)}$ to modality *i* (ref. Action selection in Section 3.4).  4:**while** true **do**  5: **for** τ←1toT **do**  6:  **Model M1:**  7:  ${\hat{X}}_{1:T}^{\left(1:2\right)},{\hat{y}}_{1:T}\leftarrow PatComClassModel1\left(x_{1:\tau }^{\left(1:2\right)}\right)$  8:  **Model M2:**  9:  ${\hat{X}}_{1:T}^{\left(1:2\right)},{\hat{y}}_{1:T}\leftarrow PatComClassModel2\left(x_{1:\tau }^{\left(1:2\right)}\right)$10:  **Model M3:**11:  ${\hat{X}}_{1:T}^{\left(1:2\right)}\leftarrow PatComClassModel1\left(x_{1:\tau }^{\left(1:2\right)}\right)$12:  ${\hat{y}}_{1:T}\leftarrow Classifier\left({\hat{X}}_{1:T}^{1:2}\right)$  **Saliency Computation** (Section 4 Action selection)13:  $S_{\tau }^{\left(1:2\right)}\leftarrow g_{1}\left(X_{\tau +1}^{\left(1:2\right)},{\hat{X}}_{\tau +1}^{\left(1:2\right)}\right)$14:  $W_{\tau }^{\left(1:2\right)}\leftarrow g_{2}\left(S_{\tau }^{\left(1:2\right)}\right)$15:  $x_{\tau +1}^{\left(1:2\right)}\leftarrow F\left(X_{\tau +1}^{\left(1:2\right)},W_{\tau }\right)$  **Learning**16:  Update {θ,ϕ} by maximizing Equations (9), (10) or (11).17: **end for**18:**end while**

**Algorithm 2** $PatComClassModel1(x_{1:\tau }^{\left(1:2\right)})$
  1:**for** t←1toT **do**  2: **Recognition Model**  3: **for** i←1to2 **do**  4:  **if** t>τ **then**  5:   $x_{t}^{\left(i\right)}\leftarrow {\hat{X}}_{t}^{\left(i\right)}$  6:  **end if**  7:  $\left[\mu_{0,t}^{\left(i\right)}\;;\sigma_{0,t}^{\left(i\right)}\right]\leftarrow \varphi^{prior}\left(h_{t-1}^{\left(i\right)}\right)$  8:  $\left[\mu_{z,t}^{\left(i\right)}\;;\sigma_{z,t}^{\left(i\right)}\right]\leftarrow \varphi^{enc}\left(\left[x_{t}^{\left(i\right)},h_{t-1}^{\left(i\right)}\right]\right)$  9: **end for** **Product of Experts**10: $z_{t}∼N\left(\mu_{0,t},\Sigma_{0,t}\right)$, where $\Sigma_{0,t}={\left(\sum_{i=1}^{2}{\Sigma_{0,t}^{\left(i\right)}}^{-2}\right)}^{-1}$ and $\mu_{0,t}=\left(\sum_{i=1}^{2}\mu_{0,t}^{\left(i\right)}{\Sigma_{0,t}^{\left(i\right)}}^{-2}\right)\Sigma_{0,t}$11: $z_{t}|x_{t}∼N\left(\mu_{z,t},\Sigma_{z,t}\right)$, where $\Sigma_{z,t}={\left(\sum_{i=1}^{2}{\Sigma_{z,t}^{\left(i\right)}}^{-2}\right)}^{-1}$ and $\mu_{z,t}=\left(\sum_{i=1}^{2}\mu_{z,t}^{\left(i\right)}{\Sigma_{z,t}^{\left(i\right)}}^{-2}\right)\Sigma_{z,t}$ **Generative Model**12: **for** i=1to2 **do**13:  $h_{t}^{\left(i\right)}\leftarrow R\;N\;N_{\theta }\left(h_{t-1}^{\left(i\right)},\left[z_{t},x_{t}^{\left(i\right)}\right]\right)$14:  $\left[\mu_{x^{\left(i\right)},t}^{\left(i\right)}\;;\sigma_{x^{\left(i\right)},t}^{\left(i\right)}\right]\leftarrow \varphi^{dec}\left(\left[h_{t-1}^{\left(i\right)},z_{t}\right]\right)$15:  ${\hat{X}}_{t}^{\left(i\right)}\leftarrow \mu_{x^{\left(i\right)},t}^{\left(i\right)}$16: **end for** **Classification Model**17: $h_{t}^{\left(3\right)}\leftarrow R\;N\;N_{\theta }\left(h_{t-1}^{\left(3\right)},\left[z_{t},x_{t},h_{t}^{\left(1\right)},h_{t}^{\left(2\right)}\right]\right)$18: ${\hat{y}}_{t}^{\left(i\right)}\leftarrow softmax\left(\left[h_{t-1}^{\left(3\right)},z_{t}\right]\right)$19:
**end for**



**Model M2.** The class label is inferred directly from partial observations, and then passed as an input to the generative model which generates the completed pattern. This is similar to the model in [49]. Mathematically,

(3) $$\begin{array}{c} \arg\;\max_{\theta }\int log\left(p_{\theta }\left(X|x_{<t},z_{\leq t}\right)p_{\theta }\left(z_{\leq t}\right)\right)dz+\arg\;\max_{\phi }\;\log q_{\phi }\left(y|x_{<t}\right) \end{array}$$

where $q_{\phi }$ is a recognition model. The model is trained end-to-end. See Figure 2b. The pseudocodes are shown in Algorithms 1 and 3.
**Algorithm 3** $PatComClassModel2(x_{1:\tau }^{\left(1:2\right)})$  1:**for** t←1toT **do**  2: **Classification Model**  3: $h_{t}^{cls}=R\;N\;N_{\alpha }^{cls}\left(h_{t-1}^{cls},x_{1:t}\right)$  4: ${\hat{y}}_{t}=softmax\left(h_{t}^{cls}\right)$  5: $h^{\prime }=tanh\left({\hat{y}}_{t}\right)$ **Recognition Model**  6: **for** i←1to2 **do**  7:  **if** t>τ **then**  8:   $x_{t}^{\left(i\right)}\leftarrow {\hat{X}}_{t}^{\left(i\right)}$  9:  **end if**10:  $\left[\mu_{0,t}^{\left(i\right)}\;;\sigma_{0,t}^{\left(i\right)}\right]\leftarrow \varphi^{prior}\left(h_{t-1}^{\left(i\right)}\right)$11:  $\left[\mu_{z,t}^{\left(i\right)}\;;\sigma_{z,t}^{\left(i\right)}\right]\leftarrow \varphi^{enc}\left(\left[x_{t}^{\left(i\right)},h_{t-1}^{\left(i\right)}\right]\right)$12: **end for**13: $\left[\mu_{0,t}^{\left(3\right)}\;;\sigma_{0,t}^{\left(i\right)}\right]\leftarrow \varphi^{prior}\left(h^{\prime }\right)$14: $\left[\mu_{z,t}^{\left(3\right)}\;;\sigma_{z,t}^{\left(i\right)}\right]\leftarrow \varphi^{enc}\left(\left[x_{t}^{\left(1\right)},x_{t}^{\left(2\right)},h^{\prime }\right]\right)$ **Product of Experts**15: $z_{t}∼N\left(\mu_{0,t},\Sigma_{0,t}\right)$, where $\Sigma_{0,t}={\left(\sum_{i=1}^{3}{\Sigma_{0,t}^{\left(i\right)}}^{-2}\right)}^{-1}$ and $\mu_{0,t}=\left(\sum_{i=1}^{3}\mu_{0,t}^{\left(i\right)}{\Sigma_{0,t}^{\left(i\right)}}^{-2}\right)\Sigma_{0,t}$16: $z_{t}|x_{t}∼N\left(\mu_{z,t},\Sigma_{z,t}\right)$, where $\Sigma_{z,t}={\left(\sum_{i=1}^{3}{\Sigma_{z,t}^{\left(i\right)}}^{-2}\right)}^{-1}$ and $\mu_{z,t}=\left(\sum_{i=1}^{3}\mu_{z,t}^{\left(i\right)}{\Sigma_{z,t}^{\left(i\right)}}^{-2}\right)\Sigma_{z,t}$ **Generative Model**17: **for** i=1to2 **do**18:  $h_{t}^{\left(i\right)}\leftarrow R\;N\;N_{\theta }\left(h_{t-1}^{\left(i\right)},\left[z_{t},x_{t}^{\left(i\right)}\right]\right)$19:  $\left[\mu_{x^{\left(i\right)},t}^{\left(i\right)}\;;\sigma_{x^{\left(i\right)},t}^{\left(i\right)}\right]\leftarrow \varphi^{dec}\left(\left[h_{t-1}^{\left(i\right)},z_{t}\right]\right)$20:  ${\hat{X}}_{t}^{\left(i\right)}\leftarrow \mu_{x^{\left(i\right)},t}^{\left(i\right)}$21: **end for**22:**end for**

**Model M3.** The completed pattern is generated from the latent variable $z_{\leq t}$. The class label is inferred from the completed pattern. The pattern completion model is pretrained:

(4) $$\arg\max_{\theta }\int log\left(p_{\theta }\left(X|x_{<t},z_{\leq t}\right)p_{\theta }\left(z_{\leq t}\right)\right)dz$$

Then the classification model is trained:

(5) $$\arg\max_{\pi }log\left(p_{\pi }\left(y|X_{<t}\right)\right)$$

Therefore, the model is not end-to-end. See Figure 2c. The pseudocodes are shown in Algorithms 1 and 2.

### 3.4. Agent Architecture

The proposed predictive agent architecture comprises five components: environment, observation, pattern completion and classification, action selection, and learning, each of which are explicated in this section. See block diagrams in Figure 1, which show the input/output relations between these components. The agent architecture is the same for the three models (M1, M2, M3) and is borrowed from [11].

1. **Environment.** The environment is the source of sensory data. It is time-varying.
2. **Observation.** The agent interacts with the environment via a sequence of eye and body movements. The observations, sampled from the environment at each time instant, are in two modalities: perceptual and proprioceptive.
3. **Pattern completion.** A multimodal variational recurrent neural network (MVRNN) for variable-length sequences is used for completing the pattern for each modality. Recognition and generation are the two processes involved in the operation of an MVRNN.

*Recognition (encoder)*. The recognition models, $q_{\phi }\left(z_{t}|x_{\leq t},z_{<t}\right)$ for models M1 and M3 and $q_{\phi }\left(z_{t}|x_{\leq t},z_{<t},y_{t}\right)$ for M2, are probabilistic encoders [46]. They produce a Gaussian distribution over the possible values of the code $z_{t}$ from which the given observations could have been generated.

**Model M1** [11]**.** The MVRNN consists of two recurrent neural networks (RNNs), each with one layer of long short-term memory (LSTM) units. Each RNN generates the parameters for the approximate posterior distribution and the conditional prior distribution for each modality, as in [50].

**Model M2.** In addition to the perceptual and proprioceptive modalities, the class label is presented as an input modality. A fully connected layer from the class labels generates the parameters for the approximate posterior density for the class modality. The recognition model generates the class label.

**Model M3.** Same as M1.

The distribution parameters from all modalities are combined using product of experts (PoE), as in [51], to generate the joint distribution parameters for both the conditional prior, $p_{\theta }\left(z_{t}|x_{<t},z_{<t}\right)$ for M1 and M3 or $p_{\theta }\left(z_{t}|x_{<t},z_{<t},y_{t}\right)$ for M2, and the approximate posterior, $q_{\phi }\left(z_{t}|x_{\leq t},z_{<t}\right)$.

The recognition model, similar to that in [50], is mathematically expressed in Lines 3–9 of Algorithm 2 and Lines 6–14 of Algorithm 3. Here, $\phi^{prior}$ generates the mean as a linear function of its input, $\phi^{enc}$ generates the logarithm of standard deviation as a nonlinear function of its input, $\phi^{prior}$ accepts the hidden state as input, and $\phi^{enc}$ accepts the hidden state and the current observation as input.

*Generation (decoder)*. **Model M1** [11]. The generative model, $p_{\theta }\left(X_{t}^{\left(1\right)},X_{t}^{\left(2\right)},y_{t}|x_{<t},z_{\leq t}\right)$, generates the perceptual and proprioceptive data and the class label from the latent variables, $z_{t}$, at each time step.

**Model M2.** The generative model, $p_{\theta }\left(X_{t}^{\left(1\right)},X_{t}^{\left(2\right)}|x_{<t},z_{\leq t}\right)$, generates the perceptual and proprioceptive data from the latent variables, $z_{t}$, at each time step.

**Model M3.** Same as M2.

Each RNN in the MVRNN generates the distribution parameters of the sensory data for a modality. The sensory data are sampled from this distribution. We assume the perceptual and proprioceptive distributions to be multivariate Gaussian as the skeletal joints are real-valued. We assume the class label distribution to be multivariate Bernoulli.

The pattern, X, is completed at each time using an iterative method. At any time *t*, the model predicts ${\hat{x}}_{t+1}$ given the observations $x_{k:t}$ (1≤k<t), then predicts ${\hat{x}}_{t+2}$ given $\left\{x_{k+1:t},{\hat{x}}_{t+1}\right\}$, then predicts ${\hat{x}}_{t+3}$ given $\left\{x_{k+2:t},{\hat{x}}_{t+1:t+2}\right\}$, and so on till ${\hat{x}}_{T}$ is predicted. This method allows a fixed and finite model to predict a variable- or infinite-length sequence. Since only the next instant is predicted at any iteration, the model can be size-efficient.

The generative model, similar to that in [50], is mathematically expressed in Lines 12–16 of Algorithm 2 and Lines 17–21 of Algorithm 3. Here, $R\;N\;N_{\theta }$ represents an LSTM unit, and $\phi^{dec}$ is the same function as $\phi^{enc}$.

4. **Action selection.** In the proposed models, action selection is to decide the weight (attention) given to each location in the environment in order to sample the current observation. At any time *t*, a saliency map $S_{t}^{\left(i\right)}$ is computed for modality *i* from which the action is determined. The saliency map assigns a salience score $S_{t,l}^{\left(i\right)}$ to each location *l*. There are 15 locations corresponding to the 15 skeleton joints: head (J1), neck (J2), torso (J3), left shoulder (J4), left elbow (J5), left hand (J6), right shoulder (J7), right elbow (J8), right hand (J9), left hip (J10), left knee (J11), left foot (J12), right hip (J13), right knee (J14), right foot (J15). As in [11], we compute the weights in three ways, as follows.

**Figure 3:**
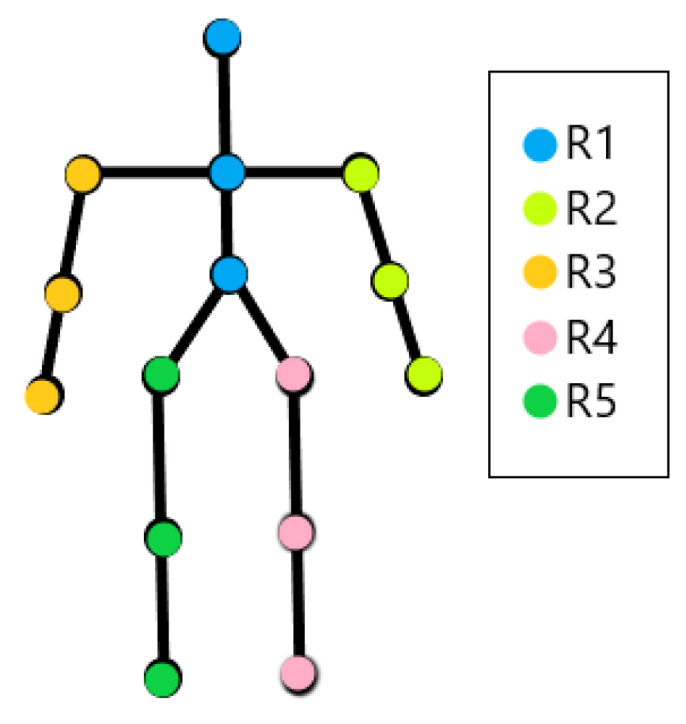
Different regions in the skeleton.

**Weights are determined by thresholding the prediction error (*pe*).** The threshold is statistically estimated on the fly and is not predetermined.

(6) $$\begin{array}{c} S_{t}^{\left(i\right)}={∥X_{t+1}^{\left(i\right)}-{\hat{X}}_{t+1}^{\left(i\right)}∥}_{1}\\ S_{t,r}^{\left(i\right)}=\frac{1}{\left|r\right|}\sum_{l\in r}S_{t,l}^{\left(i\right)}\\ W_{t,l}^{\left(i\right)}=\left\{\begin{array}{cc} 1, & \mathrm{if}\;\;S_{t,l}^{\left(i\right)}\geq \frac{1}{n_{r}}\sum_{i=1}^{n_{r}}S_{t,r}^{\left(i\right)}\\ 0, & \mathrm{otherwise} \end{array}\right.\\ x_{t+1}^{\left(i\right)}=W_{t}^{\left(i\right)}X_{t+1}^{\left(i\right)}+\left(1-W_{t}^{\left(i\right)}\right){\hat{X}}_{t+1}^{\left(i\right)} \end{array}$$

where $X_{t+1}^{\left(i\right)},{\hat{X}}_{t+1}^{\left(i\right)}$ are the true and predicted data (skeleton joint coordinates), respectively, ${∥.∥}_{1}$ denotes $L_{1}$ norm, |.| denotes the cardinality of a set, $n_{r}=5$ is the number of regions in the skeleton (J1–J3, J4–J6, J7–J9, J10–J12, J13–J15) (see Figure 3), and $S_{t,r}^{\left(i\right)}$ is the mean saliency over the joints in region *r*.

At any time, at least one region will be salient. Our experiments show that variable number of salient regions at each time step is more effective. Fixing the number of salient regions to a constant value occasionally leads to selection of regions with low saliency or overlooking regions with high saliency. In the proposed models, only the salient joints are sampled. For the nonsalient joints, the observation at time t+1 is the predicted observation from *t*.

**Weights are learned as coefficients of the prediction error (*lwpe*).**

(7) $$\begin{array}{c} S_{t}^{\left(i\right)}=W_{a}\left(X_{t+1}^{\left(i\right)}-{\hat{X}}_{t+1}^{\left(i\right)}\right)\\ W_{t}^{\left(i\right)}=\sigma \left(S_{t}^{\left(i\right)}\right)\\ x_{t+1}^{\left(i\right)}=W_{t}^{\left(i\right)}X_{t+1}^{\left(i\right)} \end{array}$$

where $W_{a}$ is the weight matrix.

**Weights are learned as coefficients of the hidden states (*lw*).**

(8) $$\begin{array}{c} S_{t}^{\left(i\right)}=W_{a}h_{t}^{\left(i\right)}\\ W_{t}^{\left(i\right)}=\sigma \left(S_{t}^{\left(i\right)}\right)\\ x_{t+1}^{\left(i\right)}=W_{t}^{\left(i\right)}X_{t+1}^{\left(i\right)} \end{array}$$

where $W_{a}$ is the weight matrix.

5. **Learning.** The objective is to maximize Equations (9)–(11) for models M1 [11], M2, M3, respectively. The derivation of these equations from the objectives of multimodal VAE [51], variational RNN [50], and VAE for classification [49] are shown in the Appendix A.

(9) $$\begin{array}{cc} E_{q_{\phi }\left(z_{\leq T}|x_{\leq T}\right)} & \left[\sum_{t=1}^{T}\sum_{i=1}^{2}\lambda_{i}\log p_{\theta }\left(X_{t}^{\left(i\right)}|z_{\leq t},x_{<t}\right)+\lambda_{3}\log p_{\theta }\left(y|z_{\leq T},x_{<T}\right)\right]\\ & -\beta \sum_{t=1}^{T}D_{KL}\left(q_{\phi }\left(z_{t}|x_{\leq t},z_{<t}\right),p_{\theta }\left(z_{t}|x_{<t},z_{<t}\right)\right) \end{array}$$

where $\lambda_{1}$, $\lambda_{2}$, $\lambda_{3}$, β are the weights balancing the terms.

(10) $$\begin{array}{c} E_{q_{\phi }\left(z_{\leq T}|x_{\leq T},y_{\leq T}\right)}\left[\sum_{t=1}^{T}\sum_{i=1}^{2}\lambda_{i}\log p_{\theta }\left(X_{t}^{\left(i\right)}|z_{\leq t},x_{<t}\right)\right]+\log p_{\theta }\left(y\right)\\ \;\;\;\;-\beta \sum_{t=1}^{T}D_{KL}\left(q_{\phi }\left(z_{t}|x_{\leq t},z_{<t},y_{t}\right),p_{\theta }\left(z_{t}|x_{<t},z_{<t},y_{t}\right)\right)+\alpha \log q_{\phi }\left(y|x_{\leq t}\right) \end{array}$$

where α controls the relative weight between generative and purely discriminative learning.

(11) $$\begin{array}{cc} E_{q_{\phi }\left(z_{\leq T}|x_{\leq T}\right)} & \left[\sum_{t=1}^{T}\sum_{i=1}^{2}\lambda_{i}\log p_{\theta }\left(X_{t}^{\left(i\right)}|z_{\leq t},x_{<t}\right)\right]\\ & -\beta \sum_{t=1}^{T}D_{KL}\left(q_{\phi }\left(z_{t}|x_{\leq t}\right),p_{\theta }\left(z_{t}\right)\right)+\log q_{\pi }\left(y|X_{1:T}\right) \end{array}$$

where $q_{\pi }\left(y|X_{1:T}\right)$ is the classification model.

## 4. Experimental Results

### 4.1. Datasets

As in [11], our models are evaluated on two datasets:

(1) The SBU Kinect Interaction Dataset [52] is a two-person interaction dataset comprising eight interactions: approaching, departing, pushing, kicking, punching, exchanging objects, hugging, and shaking hands. The data are recorded from seven participants, forming a total of 21 sets such that each set consists of a unique pair of participants performing all actions. The dataset has approximately 300 interactions of duration 9 to 46 frames. The dataset is divided into five distinct train–test splits as in [52].
(2) The K3HI: Kinect-Based 3D Human Interaction Dataset [53] is a two-person interaction dataset comprising eight interactions: approaching, departing, kicking, punching, pointing, pushing, exchanging an object, and shaking hands. The data are recorded from 15 volunteers. Each pair of participants performs all the actions. The dataset has approximately 320 interactions of duration 20 to 104 frames. The dataset is divided into four distinct train–test splits as in [53].

### 4.2. Experimental Setup

We use a single hidden layer, as in [50], for each modality in the MVRNN. Each modality in the MVRNN has a recurrent hidden layer of 256 units and a latent layer of 20 variables. These parameters are estimated empirically. *T* is variable, as interaction videos are of different lengths. Stochastic gradient descent, with a minibatch size of 100, is used to train the model. Adam optimization with a learning rate of 0.001 and default hyperparameters ($\beta_{1}=0.9$, $\beta_{2}=0.999$) are used. The objective function parameters β, $\lambda_{1}$ and $\lambda_{2}$ are fixed to 1 while $\lambda_{3}$ and α are fixed to 50. The models are trained until the error converges. To avoid overfitting, we use a dropout probability of 0.8 for M1 [11], M2, and M3 at the hidden layer for generation and 0.1 for M1 and M2 at the hidden layer for classification. All hyperparameters except the defaults are estimated from the training set by cross validation.

### 4.3. Evaluation

In the two benchmark datasets, each skeleton consists of 15 joints. The skeletal data in SBU are normalized. We do not apply any further preprocessing. We standardize the skeletal data in K3HI. Training models on low-level handcrafted features defeats the purpose of learning, hence our inclination towards operating on raw skeletal data.

Our experiments are carried out on two settings:

1. **First person:** Here we model the agent as the first person (one of the two skeletons). Its body constitutes its internal environment while the other skeleton constitutes its external (visual) environment. Two modalities are used in our model (see Figure 1a): (i) visual perception, which captures the other skeleton’s 3D joint coordinates, and (ii) body proprioception, which captures the first skeleton’s 3D joint coordinates. Here, i=1,2 in the objective function (ref. Equations (9)–(11)).
2. **Third person:** Here we model the agent as a third person (e.g., audience). The two interaction skeletons constitute the agent’s external (visual) environment. One modality is used in our model (see Figure 1b): visual perception, which captures both the skeletons’ 3D joint coordinates. Here, i=1 in the objective function (ref. Equations (9)–(11)).

**Model variations:** For each of the above two settings, we experiment with the three action selection methods (ref. “action selection” in Section 3.4): *pe*, *lwpe*, and *lw*.

**Ablation study—baseline, *bs* (without attention):** Due to lack of end-to-end models that simultaneously generate and classify two-person interactions from 3D skeletal data, our models’ performances are evaluated using an ablation study, referred to as the *baseline* (*bs*). The goal is to understand the utility of attention in our models. For that, we create a baseline model (*bs*) where attention (i.e., action selection, ref. Lines 13–15 in Algorithm 1) is eliminated from the models. The MVRNN is modified such that the observation is sampled from all the joints (i.e., weight distribution is uniform over all joints) from both the skeletons at any time. Thus, the models at any time (video frame) observe the entire skeletons.

For a fair comparison, the number of layers and number of neurons in each layer are the same over all model variants, including the baseline.

**Evaluation metrics:** We evaluate the generation accuracy using average frame distance (AFD), as in [28]: $\frac{1}{T-1}\sum_{t}{∥X_{t}^{\left(i\right)}-{\hat{X}}_{t}^{\left(i\right)}∥}^{2}$, where $X_{t}^{\left(i\right)}$ and ${\hat{X}}_{t}^{\left(i\right)}$ are the true and predicted skeletal joint coordinates, respectively, at time *t* for modality *i*, and *T* is the sequence length. We evaluate the classification performance using accuracy, recall, precision, and F1 score.

### 4.4. Evaluation Results

#### 4.4.1. Qualitative Evaluation

**Figure 4:**
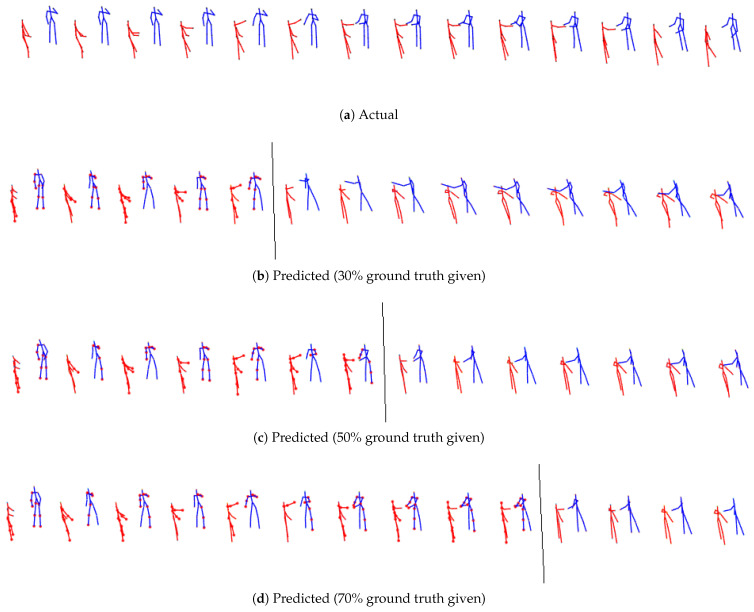
The top row represents true skeletal data for the prediction at alternate time steps for **SBU Kinect Interaction data for exchanging object for first person environment**. Each skeleton in rows 2, 3 and 4 shows one step ahead prediction until 30%, 50% and 70% of the ground truth is given (highlighted by the grey line) respectively. Beyond that, the model uses its own prediction as input for completing the patterns until the final time step is reached. The salient joints are marked red.

**Figure 5:**
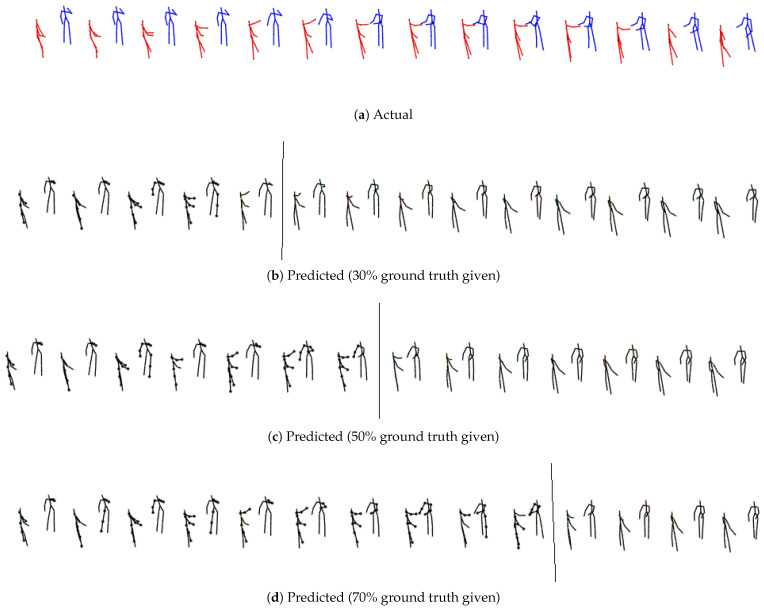
The top row represents true skeletal data for the prediction at alternate time steps for **SBU Kinect Interaction data for exchanging object for third person environment**. Each skeleton in rows 2, 3 and 4 shows one step ahead prediction until 30%, 50% and 70% of the ground truth is given (highlighted by the grey line) respectively. Beyond that, the model uses its own prediction as input for completing the patterns until the final time step is reached. The salient joints are marked red.

**Figure 6:**
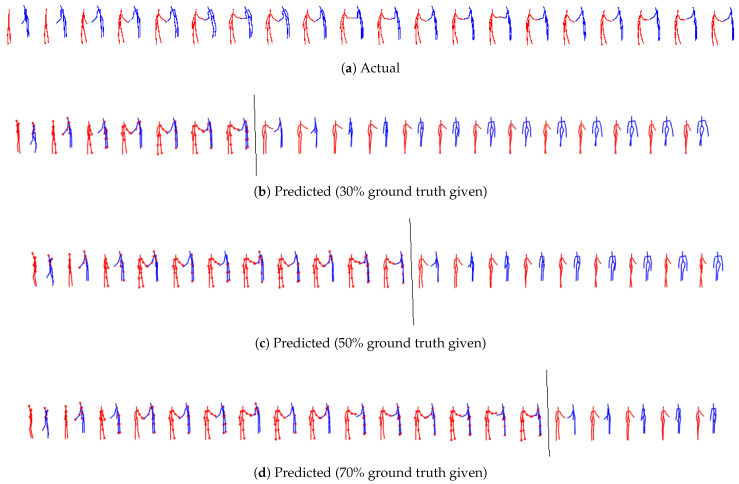
The top row represents true skeletal data for the prediction at every third instant for **K3HI Intersection data for shaking hands for first person environment**. Each skeleton in rows 2, 3 and 4 shows one step ahead prediction until 30%, 50% and 70% of the ground truth is given (highlighted by the grey line) respectively. Beyond that, the model uses its own prediction as input for completing the patterns until the final time step is reached. The salient joints are marked red.

**Figure 7:**
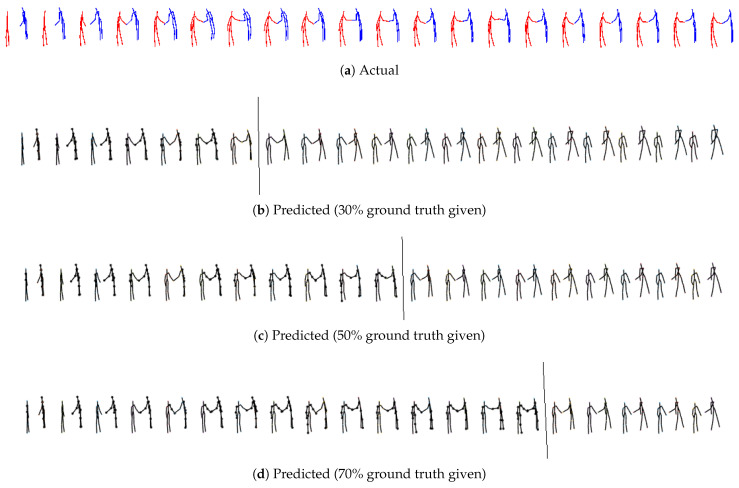
The top row represents true skeletal data for the prediction at every third instant for **K3HI Intersection data for shaking hands for third person environment**. Each skeleton in rows 2, 3 and 4 shows one step ahead prediction until 30%, 50% and 70% of the ground truth is given (highlighted by the grey line) respectively. Beyond that, the model uses its own prediction as input for completing the patterns until the final time step is reached. The salient joints are marked red.

From qualitative visualization, all three models (M1 [11], M2, M3) can generate realistic predictions over space and time for all the cases. As expected, short-term predictions are more accurate than long-term predictions. Even in the long term, there is continuity, and the two predicted skeletons are well synchronized. The proposed models’ predicted action/reaction at each time step complies with the actual interactions. See Figure 4, Figure 5, Figure 6 and Figure 7 for samples of generated interactions using M2 with *pe* action selection method.

#### 4.4.2. Evaluation for Generation Accuracy

The AFD from the first-person environment is lower than or comparable to that from the third-person for most cases (see Table 1, Table 2, Table 3 and Table 4). Modeling the two skeletons as distinct modalities helps in learning a better latent representation, resulting in more accurate generation. First-person models have more parameters than third-person models (see Table 5), which also explains the lower AFD of the first-person models.

**First person:** AFD is the lowest for *lwpe* and *bs* for the SBU Kinect dataset and *bs* for the K3HI dataset. AFD is the highest for *pe* for both datasets.

**Third person:** AFD is the lowest for *bs* for the SBU Kinect dataset and *lw* and *bs* for the K3HI dataset. AFD is the highest for *pe* for both datasets.

Within the same category for action selection, we do not observe much variation in AFD for the three models for both datasets (see Table 1, Table 2, Table 3 and Table 4). The generation (decoder) of the three models is similar, so their AFDs are comparable for any interaction class and action selection method. The generation process is more dependent on the action selection method; hence, we observe higher variation in AFD for different action selection methods (see Table 1 and Table 3).

In the proposed models (M2, M3), generation is not the primary goal but is necessary to calculate attention from generation error. That is why such attention-based models (e.g., [11,14,16]) are said to perform *recognition via generation*. M2 is unique since recognition influences generation and vice versa, while in M1 and M3, generation influences recognition but not vice versa. The models learn the spatiotemporal relations between joint locations in each skeleton using the VRNN in each modality and between the two skeletons using the PoE. M1 and M2 are learned end-to-end, while M3 is not.

#### 4.4.3. Evaluation for Classification Accuracy

In most cases, the classification accuracy of the three models (M1 [11], M2, M3) in first person is higher than or comparable to that in third person. Also, the number of trainable parameters for first-person models is greater than that of third-person models (see Table 5).

In all experiments, the top-performing attention model yields an accuracy either comparable to or higher than the baseline (*bs*). The goal of attention in our models is to foster efficiency, discussed in the next section. Also, our *bs*’s accuracy is higher than the state of the art on both datasets on raw skeleton (see Table 6).

**First person:** Among the three models, M1 yields the highest classification accuracy for almost all action selection methods for both the datasets, followed closely by M2 (see Table 7 and Table 8). Among the three action selection methods, for the SBU Kinect dataset, *bs*, *lwpe*, and *lw* yield the highest classification accuracy for M1, M2, and M3, respectively (see Table 7). For the K3HI dataset, *bs* yields the highest classification accuracy for M1 and M3, while *pe* yields the highest for M2 (see Table 8).

**Third person:** Among the three models, M1 yields the highest classification accuracy for all action selection methods for both the datasets, followed closely by M2 (see Table 9 and Table 10). Among the action selection methods, for the SBU Kinect dataset, *bs* yields the highest classification accuracy for M1 and M3, while *pe* yields the highest for M2. For the K3HI dataset, *pe* yields the highest classification accuracy for M2 and *bs* yields the highest for M1 and M3, while *lwpe* yields the lowest classification accuracy for all models.

M1 takes into account the partial observations and the latent variables for predicting the class, while M2 takes into account only the partial observations. Our results show that including the latent variables to predict the class can make a significant improvement in the classification performance. Additionally, in M1, the classification modality shares parameters with the generation modality, whereas in M2, the classification modality does not share parameters with the generation modality, though in both cases the generation modality shares parameters with the classification modality. Thus, it is possible that the generation modality improves the classification results in M1 as compared to M2. M3 uses the generated data to predict the class. As the generated skeletal data contain less discriminative features than the true skeletal data, M3’s classification accuracy is low. We did not observe a consistent pattern in the performance accuracy due to different action selection methods for the same model. Thus, no action selection method is superior to the others. Results from *pe* are comparable to or better than the baseline in all the cases for M1 and M2 (see Table 7, Table 8, Table 9 and Table 10). Results from *lwpe* and *lw* are comparable to the baseline, *bs*, for M1 and M2 for the K3HI dataset (see Table 8 and Table 10).

Table 6 compares *our* most accurate models (for different settings and action selection methods) with relevant models reported in the literature. Results show that M2 with *lwpe* for the SBU dataset and all models and action selection methods for the K3HI dataset achieve higher classification accuracy than models that operate on raw skeletal data, compared with our models.

As stated in Section 1, models that perform both generation and recognition of human–human interactions are scarce. As noted in [11], only two models, [41,42], perform generation and recognition. However, both of them solve the problem of reaction generation, while our models (M1 [11], M2, M3) solve the more challenging problem of interaction generation. Hence, results from [41,42] are not included in any of the comparison tables in this paper. In [41], classification accuracy is 80% and 46.4% for SBU and K3HI, respectively, which are much lower than our models (ref. Table 6). In [42], classification accuracy is 79.2% for aggressive emotions (kick, push, punch) and 39.97% for neutral emotions (hug, shake hands, exchange objects) for SBU, which are much lower than our models (ref. Table 6).

#### 4.4.4. Analysis of Action Selection

We can visualize the distribution of attention weights assigned to the joints or regions as a heatmap (see Figure A1, Figure A2, Figure A3, Figure A4, Figure A5, Figure A6, Figure A7, Figure A8, Figure A9, Figure A10, Figure A11 and Figure A12 in the Appendix B). For each interaction class, this distribution over the joints/regions is computed from the sum of all weights $W_{t}$ (ref. Equations (6)–(8)) assigned to each joint/region.

**Figure A1:**
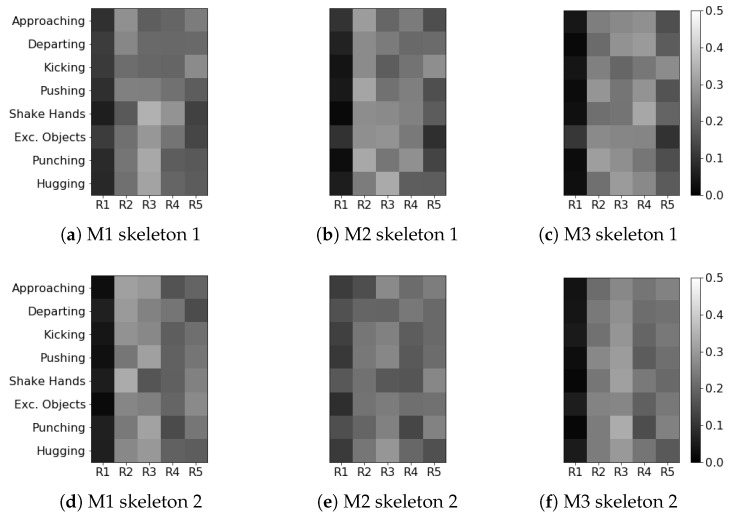
Salient region distribution (dist.) over all interactions shown for skeleton 1 in (**a**–**c**) and the other skeleton in (**d**–**f**) for **first person, (*pe*)** environment using SBU Kinect interaction data.

**Figure A4:**
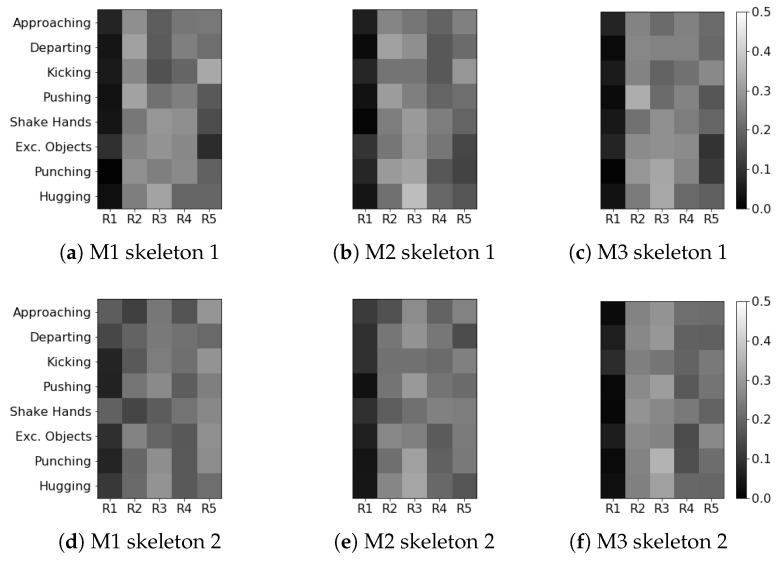
Salient region distribution (dist.) over all interactions shown for skeleton 1 in (**a**–**c**) and the other skeleton in (**d**–**f**) for **third person, (*pe*)** environment using SBU Kinect interaction data.

**Figure A7:**
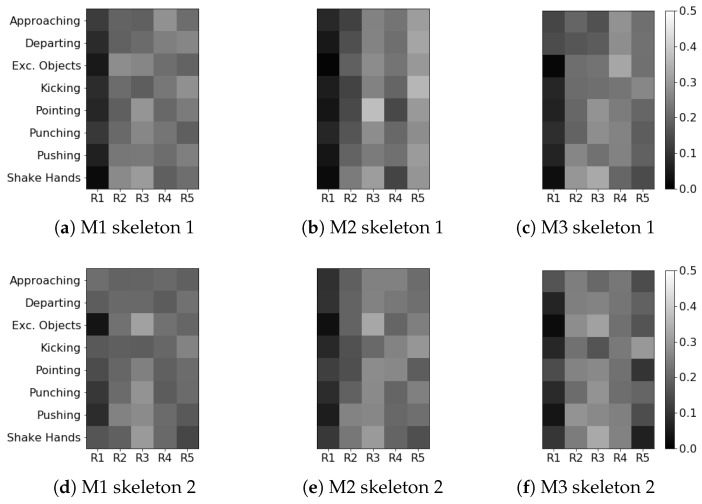
Salient region distribution (dist.) over all interactions shown for skeleton 1 in (**a**–**c**) and the other skeleton in (**d**–**f**) for **first person, (*pe*)** environment using K3HI interaction data.

**Figure A10:**
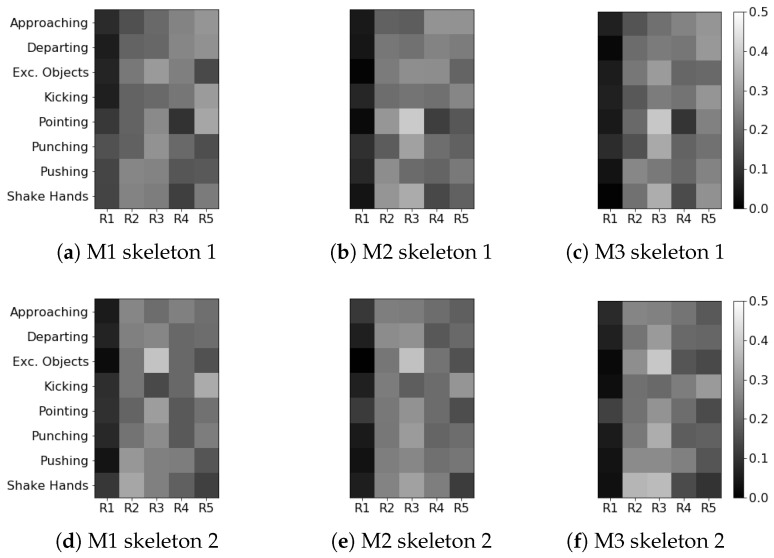
Salient region distribution (dist.) over all interactions shown for skeleton 1 in (**a**–**c**) and the other skeleton in (**d**–**f**) for **third person, (*pe*)** environment using K3HI interaction data.

The joints, whose movements have high variation over time, are more difficult to predict and hence are more salient. Thus, the salient regions for punch, exchange objects, push, handshake, and hug are primarily the hands (e.g., punch in Figure A1c and Figure A4b; exchange object in Figure A7a,f; push in Figure A7d; shake hands in Figure A10b,f; hug in Figure A4a,e and Figure A1b,d), while for kicking, they are the legs (ref. Figure A7e and Figure A10f). This is not observed in some cases, such as kicking in Figure A1d, because the same skeleton might be the interaction initiator in some videos and the reactor in the others within the same dataset, thereby having different behaviors for the same interaction class.

We do not observe much variation in the distributions between M1, M2, M3 for the same action selection method. For any interaction, the weight distributions from *lwpe* and *lw* are similar. The attention weights are not very different for the different interactions.

#### 4.4.5. Evaluation for Efficiency

**Figure 8:**
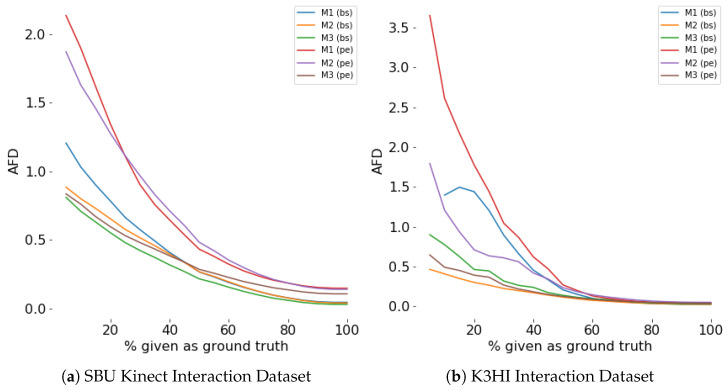
Prediction (AFD) for different percentage of ground truth given as input for **first person**. For any percentage *p*, p% of the actual data is given as input and the prediction is considered as input for the rest of the time steps.

**Figure 9:**
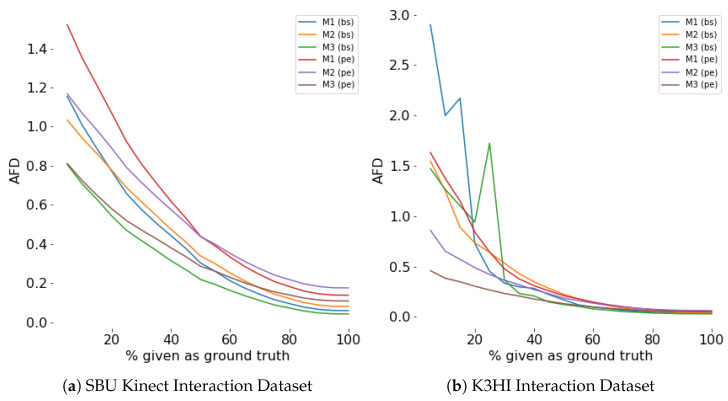
Prediction (AFD) for different percentage of ground truth given as input for **third person**. For any percentage *p*, p% of the actual data is given as input and the prediction is considered as input for the rest of the time steps.

**Figure 10:**
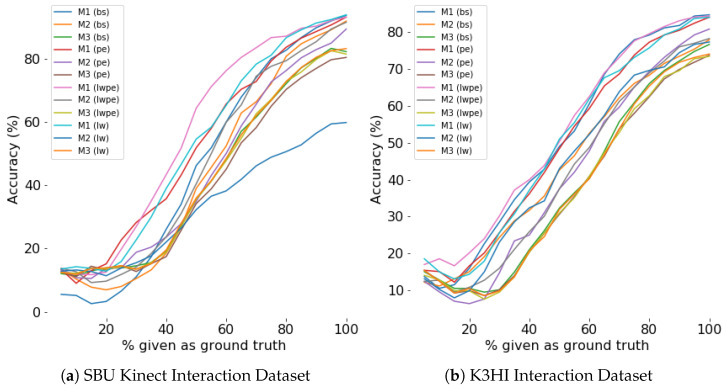
Classification accuracy for different percentage of ground truth given as input for **first person**. For any percentage *p*, p% of the actual data is given as input and the prediction is considered as input for the rest of the time steps.

**Figure 11:**
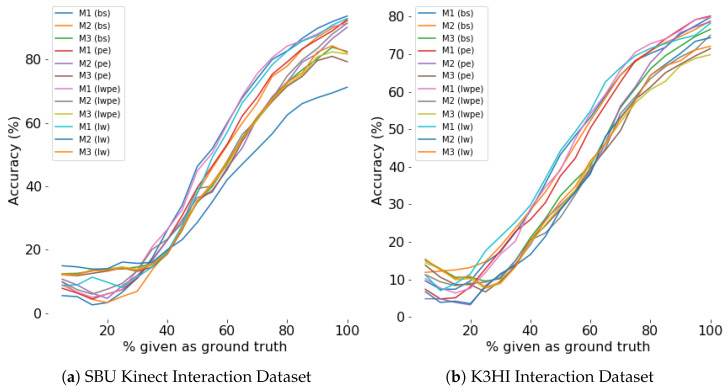
Classification accuracy for different percentage of ground truth given as input for **third person**. For any percentage *p*, p% of the actual data is given as input and the prediction is considered as input for the rest of the time steps.

Efficiency of a model is evaluated by the percentage of the scene observed for prediction. Our experiments show that during the first few sampling instants, both generation and classification accuracy improves exponentially (see Figure 8, Figure 9, Figure 10 and Figure 11). The saturation of the accuracy after that indicates our models do not need to sample a larger percentage of the data as ground truth for generation.

We compute the average (over all videos for each interaction) of the number of salient joints sampled by our models at each glimpse (see Table 11 and Table 12). We do not observe much variation in the average percentage for different models for both the datasets and for first- and third-person environments. On average, for any interaction in the two datasets, our model samples less than 49% and 48% of the joints in the case of FP and TP, respectively. For both datasets, the highest sparsity is for kicking. The lowest sparsity is for punching (FP) and punching/pushing (TP) for the SBU Kinect dataset and approaching/exchange object (FP) and approaching/departing (TP) for the K3HI dataset.

### 4.5. Design Evaluation for Different Models

#### 4.5.1. Handling Missing Class Labels

The three models (M1 [11], M2, M3) require true class labels to train for classification. A subset of parameters in each model is shared between the classification and generation pathways, albeit in unique ways (see Figure 2). In M1, the generation (completed pattern) and class label are independent outputs. In M2, the class label is an input to the generative pathway; hence, classification accuracy directly influences generation accuracy. In M3, the completed pattern is the input to the classification model; hence, generation accuracy directly influences classification accuracy.

When class labels are missing, the generative parameters, including the shared parameters, are trained to minimize the generative loss only. All three models continue to infer irrespective of whether labels are present, noisy, or missing, which makes them practical for real-world applications. A drawback of M2 is that the generation depends on the predicted class label; hence, the generation will be poor if the classification pathway is not well trained. An advantage of M1 and M3 is that because the generation and classification pathways share parameters, even if the class labels are missing, the shared parameters will be updated by minimizing the generative error only, which might improve the classification accuracy.

#### 4.5.2. Number of Trainable Parameters

The number of trainable parameters for the three models is shown in Table 5. Third-person models have fewer trainable parameters. M1 has the most and M3 has the fewest trainable parameters. *lwpe* and *lw* have more trainable parameters than *pe* or *bs*.

#### 4.5.3. Training Time

The three models (M1 [11], M2, M3) are implemented using the TensorFlow 1.3 framework in Python 3.5.4. All experiments are carried out in a high-performance computing (HPC) facility using PowerEdge R740 GPU nodes equipped with Tesla V100-PCIE-16GB.

Training time is the time required to train a model on the training set until the error converges. The training time for our models is shown in Table 13, where we report the average (over n-fold cross validation) convergence time in hours and the average number of iterations. In order to identify offline the iteration at which convergence occurs, we smooth the classification accuracy and the generation error curves by calculating the moving average with a 50-iteration window. For classification, we assume convergence is reached at the iteration when the average accuracy exceeds 90% of the highest accuracy for M1, M2, and M3. When pretraining M3’s generative model, convergence is reached at the iteration when the average error falls below 10% of the highest error.

For the SBU Kinect dataset and both first-person and third-person environments, M3 and M2 require the least and highest training times for all action selection methods. For the K3HI dataset, M3 requires the least training time for all action selection methods for both environments, and M2 requires the highest training time for all action selection methods except M1 (*pe*) for first person.

M3 is trained separately for generation and classification, while M1 and M2 are trained for generation and classification jointly. Thus, the model trained for a single task converges faster than the models trained jointly for multiple tasks.

#### 4.5.4. End-to-End Training

End-to-end training allows an entire model to be optimized for a given task(s) and dataset. However, the challenge is to search for the optimal set of parameter values in a very large space. This is often circumvented by *pretraining* selected components (layers, blocks, functions) in isolation for a number of iterations to initialize their parameters in a suboptimal space. Then the entire model is trained end-to-end. In this paper, models M1 and M2 are trained end-to-end without any pretraining, while M3 is not end-to-end.

## 5. Discussion

This section discusses the limitations of the proposed approach for human–human interaction recognition via generation and also discusses future work.

### 5.1. Limitations of the Proposed Approach

The limitations stated below apply to the proposed approach and to almost all related works.

#### 5.1.1. Limited Interaction Context

The physical interaction between two humans can be influenced by a wide range of variables such as age, gender, culture, personality, style, mood, relationship, context (e.g., formal vs. informal setting), difference in socioeconomic status, health, disability, past experiences (especially traumatic ones), social norms, and state of physical environment (e.g., crowded vs. open). Accounting for these variables is essential for understanding human–human interactions and developing interactive systems that can perform effectively across diverse scenarios. These variables have not been explicitly considered in the proposed approach and related works. However, the approaches that learn by imitation, such as ours, do implicitly consider some of these variables if they are captured in the training data.

#### 5.1.2. Limited Interaction Modalities

Humans interact by the simultaneous use of multiple modalities such as text, speech, nonspeech vocalizations (e.g., sigh, laughter, murmur), facial expressions, gaze and eye contact, body movements for gestures and touch, proxemics, and olfactory cues, which convey emotions and intentions. The proposed approach and related works have largely concentrated only on body movements to infer intent.

#### 5.1.3. Need for Labeled Training Data

The proposed approach and related works on interaction recognition are trained using data labeled with class labels. Given that labeled data are scarce and unlabeled data are abundant, it is imperative to develop models that can learn from unlabeled data.

### 5.2. Future Work

Our future work is to address the limitations of the proposed approach stated above and to make the approach more accurate and versatile.

#### 5.2.1. Incorporate More Interaction Context

Incorporating interaction context in an AI model requires data about the context. Such data are scarce, primarily due to restrictions on usage of soft and hard sensors to collect data due to risk of confidentiality breach and privacy invasion. An alternative is to generate data using a combination of physics-based and generative AI models (see [61], for example).

#### 5.2.2. Incorporate Multiple Interaction Modalities

Incorporating multiple interaction modalities would lead to more robust inference of the interacting human’s intentions and emotions, which would help to generate more effective reactions. The proposed model is inherently multimodal. It combines multiple modalities using PoE, which is a scalable approach as the number of parameters increases linearly with the number of modalities *m*. All multimodal models are not linearly scalable. For example, the Multimodal Transformer (MulT) [62] learns a mapping from each modality to another, thereby learning $O(m^{2})$ mappings. As a result, the number of parameters increases quadratically with the number of modalities. The proposed model can be extended to incorporate multiple modalities in a relatively simple manner and has already been tested on different kinds of signals, such as body/skeletal motion [11,15] (and this current article), images and videos [12,13,14], and speech [16].

#### 5.2.3. Alleviate the Need for Labeled Training Data

There are multiple ways to train a classifier with data not labeled with class labels. These include unsupervised learning methods (e.g., clustering, anomaly detection, non-negative matrix factorization, autoencoder), semisupervised learning methods (utilize a small amount of labeled data along with a large amount of unlabeled data), and self-supervised learning methods (learn representations from the unlabeled data by solving a pretext task, such as predicting the next word in a sequence or reconstructing the input, followed by fine-tuning on a small amount of labeled data for the target classification task). The proposed model can be easily trained using semi-supervised or self-supervised methods.

#### 5.2.4. Experiment with Other Models

**Figure 12:**
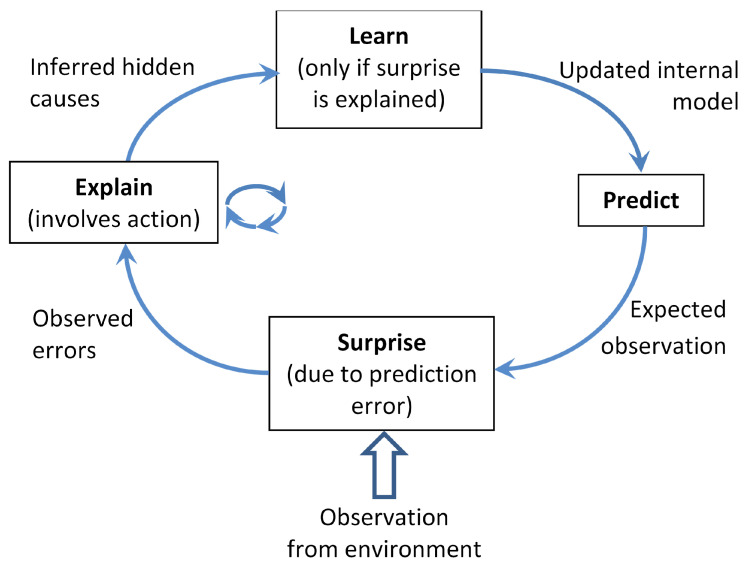
Block diagram of the SELP cycle [63,64], which forms the basis of the proposed agent model and related agent models [11,13,14,15,16,65,66,67,68].

In our earlier works [63,64], a general-purpose predictive agent was proposed that interacts with its environment by relentlessly executing four functions cyclically: **S**urprise (compute prediction error), **E**xplain (infer causes of surprise), **L**earn (update internal model using the surprise and inferred causes), and **P**redict the next observation (see Figure 12). In order to **E**xplain, the agent can act, which includes interaction and communication with its own body (sensed via proprioception) and with its environment and other agents (sensed via perception). The proposed agent architecture (ref. Figure 1) is an implementation of the SELP cycle, which is modular and allows experimentation with different generative models in place of VAE or VRNN, and different fusion methods in place of PoE. It is interesting to note that our earlier works [65,66] proposed an agent model that decide when and with whom to communicate/interact, while the agent model proposed in this current work (and [11]) propose how to interact, all following the SELP cycle.

## 6. Conclusions

Two agent models are proposed that sequentially sample and interact with their environment, which is constituted by 3D skeletons. At each instant, they sample a subset of skeleton joints to jointly minimize their classification and sensory prediction (or generation) errors in a greedy manner. The agents operate as closed-loop systems involving perceptual (“what”) and proprioceptive (“where”) pathways. One of the proposed agent models is learned end-to-end, while the other is not. Extensive experiments on interaction classification and generation on benchmark datasets in comparison with a state-of-the-art model reveal that one of the proposed models is more size-efficient but still yields classification and generation accuracy comparable to the state of the art. Interesting insights drawn from the design of these models will be useful for designing efficient generative AI (GenAI) systems. The future of AI is agents. Our agent models consisting of perceptual and proprioceptive pathways in a multimodal setting contribute a unique idea towards the development of AI agents.

## Figures and Tables

**Table 1:** Generation accuracy (AFD) averaged over all examples for each interaction in the test set and all train–test splits (mean, std. dev.) for **first-person** environment for **SBU Kinect Interaction Dataset**. (*bs*), (*pe*), (*lwpe*), and (*lw*) are different action selection methods (ref. Section 4, action selection). Interactions of approach, shake hands, and exchange object are abbreviated as Appr, Sh Hands, and Exc Obj, respectively. Average is abbreviated as Avg.

Model	Appr	Depart	Kick	Push	Sh Hands	Hug	Exc Obj	Punch	Avg AFD
M1 (*bs*)	0.031, 0.02	0.034, 0.02	0.072, 0.04	0.044, 0.02	0.032, 0.01	0.060, 0.02	0.037, 0.05	0.053, 0.02	0.045, 0.01
M2 (*bs*)	0.026, 0.01	0.028, 0.02	0.064, 0.03	0.043, 0.02	0.031, 0.02	0.055, 0.02	0.032, 0.01	0.046, 0.02	0.041, 0.01
M3 (*bs*)	0.020, 0.01	0.023, 0.02	0.050, 0.03	0.030, 0.01	0.021, 0.01	0.042, 0.02	0.024, 0.01	0.036, 0.02	**0.031, 0.01**
M1 (*pe*)	0.102, 0.07	0.125, 0.10	0.244, 0.27	0.129, 0.10	0.112, 0.06	0.171, 0.11	0.132, 0.10	0.170, 0.11	0.148, 0.04
M2 (*pe*)	0.092, 0.06	0.100, 0.07	0.228, 0.20	0.131, 0.08	0.113, 0.06	0.170, 0.07	0.126, 0.11	0.159, 0.11	0.140, 0.04
M3 (*pe*)	0.065, 0.05	0.085, 0.06	0.189, 0.28	0.093, 0.10	0.076, 0.03	0.129, 0.07	0.092, 0.10	0.126, 0.12	**0.107, 0.04**
M1 (*lwpe*)	0.028, 0.02	0.033, 0.02	0.071, 0.04	0.043, 0.02	0.032, 0.03	0.059, 0.03	0.035, 0.01	0.052, 0.02	0.044, 0.01
M2 (*lwpe*)	0.029, 0.02	0.033, 0.02	0.077, 0.04	0.046, 0.02	0.033, 0.03	0.062, 0.02	0.036, 0.01	0.056, 0.02	0.047, 0.02
M3 (*lwpe*)	0.026, 0.02	0.030, 0.02	0.067, 0.04	0.040, 0.02	0.027, 0.02	0.052, 0.02	0.033, 0.02	0.047, 0.02	**0.040, 0.01**
M1 (*lw*)	0.032, 0.02	0.035, 0.02	0.072, 0.04	0.045, 0.02	0.032, 0.02	0.057, 0.02	0.036, 0.02	0.052, 0.02	0.045, 0.01
M2 (*lw*)	0.061, 0.05	0.066, 0.07	0.146, 0.10	0.102, 0.05	0.076, 0.06	0.125, 0.07	0.082, 0.05	0.113, 0.07	0.096, 0.03
M3 (*lw*)	0.020, 0.01	0.023, 0.02	0.052, 0.03	0.031, 0.02	0.022, 0.01	0.043, 0.02	0.025, 0.01	0.037, 0.02	**0.032, 0.01**

**Table 2:** Generation accuracy (AFD) averaged over all examples for each interaction in the test set and all train–test splits (mean, std dev) for **third-person** environment for **SBU Kinect Interaction Dataset**. (*bs*), (*pe*), (*lwpe*), and (*lw*) are different action selection methods (ref. Section 4, action selection); interactions of approach, shake hands, and exchange object are abbreviated as Appr., Sh. Hands, and Exc. ob., respectively; metric average AFD is abbreviated as Avg. AFD.

Model	Appr.	Depart	Kick	Push	Sh. Hands	Hug	Exc. Ob.	Punch	Avg. AFD
M1 (*bs*)	0.040, 0.03	0.043, 0.03	0.097, 0.05	0.059, 0.03	0.042, 0.03	0.075, 0.04	0.046, 0.01	0.067, 0.03	0.059, 0.02
M2 (*bs*)	0.056, 0.04	0.058, 0.04	0.134, 0.08	0.083, 0.04	0.056, 0.05	0.100, 0.05	0.063, 0.02	0.092, 0.05	0.080, 0.03
M3 (*bs*)	0.026, 0.02	0.030, 0.02	0.072, 0.04	0.042, 0.02	0.028, 0.02	0.056, 0.02	0.034, 0.01	0.049, 0.02	**0.042, 0.02**
M1 (*pe*)	0.098, 0.04	0.101, 0.04	0.215, 0.08	0.114, 0.07	0.172, 0.07	0.108, 0.04	0.152, 0.04	0.152, 0.04	0.137, 0.04
M2 (*pe*)	0.118, 0.06	0.129, 0.06	0.279, 0.11	0.171, 0.08	0.126, 0.08	0.215, 0.06	0.126, 0.04	0.186, 0.04	0.169, 0.06
M3 (*pe*)	0.068, 0.04	0.079, 0.04	0.184, 0.07	0.107, 0.04	0.082, 0.04	0.141, 0.06	0.082, 0.03	0.120, 0.03	**0.108, 0.04**
M1 (*lwpe*)	0.046, 0.04	0.054, 0.05	0.121, 0.06	0.072, 0.03	0.051, 0.03	0.095, 0.04	0.059, 0.02	0.083, 0.03	0.073, 0.02
M2 (*lwpe*)	0.078, 0.06	0.084, 0.09	0.177, 0.10	0.108, 0.04	0.079, 0.04	0.144, 0.08	0.089, 0.04	0.133, 0.07	0.111, 0.04
M3 (*lwpe*)	0.038, 0.03	0.044, 0.03	0.095, 0.05	0.055, 0.02	0.039, 0.04	0.073, 0.03	0.046, 0.02	0.065, 0.02	**0.057, 0.02**
M1 (*lw*)	0.042, 0.03	0.047, 0.03	0.108, 0.07	0.063, 0.03	0.044, 0.04	0.077, 0.04	0.048, 0.01	0.071, 0.03	0.062, 0.02
M2 (*lw*)	0.076, 0.09	0.119, 0.22	0.191, 0.18	0.124, 0.10	0.092, 0.08	0.155, 0.14	0.101, 0.10	0.139, 0.11	0.125, 0.04
M3 (*lw*)	0.028, 0.02	0.033, 0.02	0.078, 0.04	0.042, 0.02	0.029, 0.02	0.057, 0.02	0.034, 0.01	0.050, 0.02	**0.044, 0.02**

**Table 3:** Generation accuracy (AFD) averaged over all examples for each interaction in the test set and all train–test splits (mean, std dev) for **first-person** environment for **K3HI Interaction Dataset**. (*bs*), (*pe*), (*lwpe*), and (*lw*) are different action selection methods (ref. Section 4, action selection); interactions of approach, shake hands, and exchange object are abbreviated as Appr., Sh. Hands, and Exc. ob., respectively; metric average AFD is abbreviated as Avg. AFD.

Model	Appr.	Depart	Exc. Ob.	Kick	Point	Punch	Push	Sh. Hands	Avg. AFD
M1 (*bs*)	0.153, 0.99	0.015, 0.01	0.006, 0.01	0.011, 0.01	0.007, 0.00	0.010, 0.01	0.010, 0.00	0.006, 0.00	**0.027, 0.05**
M2 (*bs*)	0.146, 1.0	0.016, 0.01	0.006, 0.01	0.012, 0.01	0.008, 0.00	0.010, 0.01	0.010, 0.01	0.006, 0.00	**0.027, 0.05**
M3 (*bs*)	0.143, 0.85	0.022, 0.02	0.013, 0.01	0.022, 0.03	0.016, 0.02	0.020, 0.03	0.019, 0.02	0.012, 0.02	0.033, 0.04
M1 (*pe*)	0.135, 0.74	0.037, 0.03	0.020, 0.01	0.033, 0.02	0.025, 0.02	0.026, 0.02	0.027, 0.01	0.019, 0.02	**0.040, 0.04**
M2 (*pe*)	0.136, 0.66	0.048, 0.03	0.029, 0.02	0.052, 0.03	0.038, 0.03	0.039, 0.02	0.041, 0.02	0.031, 0.02	0.052, 0.03
M3 (*pe*)	0.126, 0.61	0.041, 0.03	0.021, 0.01	0.038, 0.03	0.028, 0.03	.029, 0.03	0.031, 0.02	0.021, 0.02	0.042, 0.03
M1 (*lwpe*)	0.143, 0.87	0.017, 0.02	0.007, 0.01	0.013, 0.01	0.010, 0.02	0.011, 0.01	0.011, 0.01	0.007, 0.01	**0.027, 0.05**
M2 (*lwpe*)	0.148, 0.91	0.020, 0.02	0.009, 0.01	0.016, 0.01	0.012, 0.01	0.013, 0.01	0.013, 0.01	0.009, 0.01	0.030, 0.05
M3 (*lwpe*)	0.135, 0.75	0.029, 0.03	0.017, 0.02	0.031, 0.05	0.021, 0.03	0.026, 0.04	0.027, 0.04	0.017, 0.03	0.038, 0.04
M1 (*lw*)	0.164, 1.1	0.016, 0.01	0.006, 0.01	0.012, 0.01	0.007, 0.00	0.009, 0.01	0.009, 0.01	0.006, 0.00	**0.029, 0.05**
M2 (*lw*)	0.154, 0.97	0.018, 0.02	0.007, 0.01	0.014, 0.01	0.008, 0.01	0.011, 0.01	0.011, 0.01	0.006, 0.01	**0.029, 0.05**
M3 (*lw*)	0.141, 0.85	0.027, 0.02	0.017, 0.02	0.030, 0.05	0.021, 0.03	0.026, 0.04	0.025, 0.04	0.017, 0.03	0.038, 0.04

**Table 4:** Generation accuracy (AFD) averaged over all examples for each interaction in the test set and all train–test splits (mean, std dev) for **third-person** environment for **K3HI Interaction Dataset**. (*bs*), (*pe*), (*lwpe*), and (*lw*) are different action selection methods (ref. Section 4, action selection); interactions of approach, shake hands, and exchange object are abbreviated as Appr., Sh. Hands, and Exc. ob., respectively; metric average AFD is abbreviated as Avg. AFD.

Model	Appr.	Depart	Exc. Ob.	Kick	Point	Punch	Push	Sh. Hands	Avg. AFD
M1 (*bs*)	0.155, 0.96	0.024, 0.01	0.013, 0.01	0.025, 0.02	0.018, 0.02	0.019, 0.02	0.020, 0.01	0.014, 0.01	0.036, 0.05
M2 (*bs*)	0.155, 0.89	0.026, 0.01	0.016, 0.01	0.027, 0.02	0.023, 0.03	0.022, 0.02	0.023, 0.01	0.019, 0.02	0.039, 0.05
M3 (*bs*)	0.154, 0.96	0.017, 0.01	0.007, 0.01	0.015, 0.01	0.010, 0.01	0.011, 0.01	0.012, 0.01	0.007, 0.01	**0.029, 0.05**
M1 (*pe*)	0.161, 0.75	0.044, 0.02	0.027, 0.02	0.054, 0.03	0.047, 0.04	0.040, 0.02	0.042, 0.02	0.031, 0.02	0.056, 0.04
M2 (*pe*)	0.169, 0.66	0.047, 0.02	0.031, 0.02	0.062, 0.03	0.055, 0.05	0.046, 0.02	0.048, 0.02	0.035, 0.02	0.062, 0.04
M3 (*pe*)	0.154, 0.71	0.038, 0.02	0.024, 0.02	0.048, 0.03	0.038, 0.03	0.037, 0.03	0.039, 0.02	0.026, 0.02	**0.051, 0.04**
M1 (*lwpe*)	0.159, 0.94	0.024, 0.02	0.013, 0.01	0.026, 0.02	0.022, 0.03	0.019, 0.01	0.021, 0.01	0.014, 0.01	**0.037, 0.05**
M2 (*lwpe*)	0.156, 0.92	0.029, 0.02	0.020, 0.02	0.036, 0.03	0.029, 0.03	0.029, 0.02	0.031, 0.02	0.020, 0.01	0.044, 0.04
M3 (*lwpe*)	0.151, 1.0	0.033, 0.02	0.021, 0.02	0.041, 0.05	0.039, 0.05	0.033, 0.03	0.033, 0.03	0.023, 0.02	0.047, 0.04
M1 (*lw*)	0.161, 1.0	0.021, 0.02	0.010, 0.01	0.020, 0.01	0.015, 0.02	0.014, 0.01	0.015, 0.01	0.009, 0.01	**0.033, 0.05**
M2 (*lw*)	0.154, 0.92	0.024, 0.02	0.012, 0.01	0.024, 0.02	0.019, 0.02	0.018, 0.01	0.019, 0.01	0.012, 0.01	0.035, 0.05
M3 (*lw*)	0.146, 0.90	0.031, 0.02	0.019, 0.02	0.036, 0.05	0.030, 0.04	0.030, 0.04	0.030, 0.04	0.020, 0.03	0.043, 0.03

**Table 5:** Number of trainable parameters.

Model	First Person	Third Person
M1 (*bs*) [11]	1,656,348	1,089,996
M2 (*bs*)	1,134,284	833,676
M3 (*bs*)	1,111,420	827,692
M1 (*pe*) [11]	1,656,348	1,089,996
M2 (*pe*)	1,134,284	833,676
M3 (*pe*)	1,111,420	827,692
M1 (*lwpe*) [11]	1,657,728	1,092,726
M2 (*lwpe*)	1,135,664	836,406
M3 (*lwpe*)	1,112,800	830,422
M1 (*lw*) [11]	1,657,728	1,092,726
M2 (*lw*)	1,135,664	836,406
M3 (*lw*)	1,112,800	830,422

**Table 6:** Comparison of classification accuracy. In the table, “our models” refers to the three models (M1, M2, M3) discussed in this paper, even though M1 was proposed in [11]. The other models cited in this table ([53,54,55,56,57,58,59,60]) perform classification only (no generation). They take both skeletons as input, similar to our models. These works do not distinguish between first- and third-person environments.

Dataset	Characteristics			Models		Accuracy
	Raw Skeleton	Skeletal Features	Attention			
SBU		✓		Other models	[54]	96.3
		✓			[55]	94.12
		✓			[56]	94.28
	✓				[57]	90.41
	✓				[58]	93.3
	✓				[57,59]	80.35
	✓			Our models (first person)	M1 (*bs*)	93.2
	✓		✓		M1 (*pe*)	93.1
	✓		✓		M2 (*lwpe*)	93.8
	✓		✓		M1 (*lw*)	91.5
	✓			Our models (third person)	M1 (*bs*)	93.7
	✓		✓		M1 (*pe*)	92.5
	✓		✓		M2 (*lwpe*)	91.4
	✓		✓		M1 (*lw*)	92.9
K3HI		✓		Other models	[53]	83.33
		✓			[60]	80.87
	✓				[53]	45.2
	✓				[60]	48.54
	✓			Our models (first person)	M1 (*bs*)	87.5
	✓		✓		M1 (*pe*)	85.9
	✓		✓		M2 (*lwpe*)	84.9
	✓		✓		M1 (*lw*)	86.9
	✓			Our models (third person)	M1 (*bs*)	83.0
	✓		✓		M1 (*pe*)	82.7
	✓		✓		M2 (*lwpe*)	82.1
	✓		✓		M1 (*lw*)	80.8

**Table 7:** Class prediction results using **first-person** environment and **SBU Kinect Interaction Dataset**. (*bs*), (*pe*), (*lwpe*), and (*lw*) are different action selection methods (ref. Section 4, action selection); classification accuracy is abbreviated as Acc.

Model	Acc.	Recall	Precision	F1 Score
M1 (*bs*)	**93.2, 4.7**	**0.934, 0.04**	**0.931, 0.05**	**0.928, 0.05**
M2 (*bs*)	91.9, 5.6	0.927, 0.04	0.913, 0.06	0.912, 0.05
M3 (*bs*)	82.2, 10.1	0.846, 0.09	0.817, 0.11	0.814, 0.11
M1 (*pe*)	**93.1, 3.75**	**0.940, 0.03**	**0.924, 0.04**	**0.925, 0.03**
M2 (*pe*)	89.3, 5.1	0.895, 0.03	0.869, 0.05	0.886, 0.04
M3 (*pe*)	80.4, 8.5	0.837, 0.08	0.799, 0.09	0.796, 0.09
M1 (*lwpe*)	93.1, 3.9	0.939, 0.04	0.929, 0.04	0.929, 0.04
M2 (*lwpe*)	**93.8, 4.7**	**0.945, 0.04**	**0.934, 0.06**	**0.931, 0.06**
M3 (*lwpe*)	81.4, 9.1	0.842, 0.08	0.809, 0.10	0.807, 0.10
M1 (*lw*)	**91.5, 6.0**	**0.920, 0.05**	**0.902, 0.07**	**0.903, 0.07**
M2 (*lw*)	59.8, 14.7	0.655, 0.13	0.564, 0.14	0.627, 0.13
M3 (*lw*)	83.2, 8.3	0.855, 0.07	0.823, 0.09	0.823, 0.09

**Table 8:** Class prediction results using **first-person** environment and **K3HI Interaction Dataset**. (*bs*), (*pe*), (*lwpe*), and (*lw*) are different action selection methods (ref. Section 4, action selection); classification accuracy is abbreviated as Acc.

Model	Acc.	Recall	Precision	F1 Score
M1 (*bs*)	**87.5, 7.1**	**0.865, 0.08**	**0.859, 0.08**	**0.856, 0.08**
M2 (*bs*)	82.7, 3.1	0.817, 0.04	0.806, 0.04	0.804, 0.04
M3 (*bs*)	80.1, 3.1	0.796, 0.03	0.783, 0.02	0.777, 0.03
M1 (*pe*)	**85.9, 5.2**	**0.854, 0.07**	**0.838, 0.06**	**0.839, 0.06**
M2 (*pe*)	84.9, 3.3	0.836, 0.04	0.835, 0.04	0.831, 0.04
M3 (*pe*)	76.9, 2.6	0.768, 0.02	0.760, 0.02	0.752, 0.02
M1 (*lwpe*)	**84.9, 3.5**	**0.850, 0.05**	**0.818, 0.03**	**0.818, 0.03**
M2 (*lwpe*)	82.1, 6.3	0.828, 0.07	0.802, 0.07	0.801, 0.06
M3 (*lwpe*)	75.6, 4.0	0.759, 0.03	0.746, 0.03	0.739, 0.03
M1 (*lw*)	**86.9, 4.3**	**0.865, 0.05**	**0.852, 0.05**	**0.853, 0.05**
M2 (*lw*)	83.7, 3.0	0.840, 0.05	0.824, 0.04	0.822, 0.04
M3 (*lw*)	76.3, 4.7	0.760, 0.04	0.753, 0.04	0.745, 0.04

**Table 9:** Class prediction results using **third-person** environment and **SBU Kinect Interaction Dataset**. (*bs*), (*pe*), (*lwpe*), and (*lw*) are different action selection methods (ref. Section 4, action selection); classification accuracy is abbreviated as Acc.

Model	Acc.	Recall	Precision	F1 Score
M1 (*bs*)	**93.7, 6.1**	**0.944, 0.05**	**0.935, 0.05**	**0.934, 0.06**
M2 (*bs*)	92.1, 3.9	0.923, 0.03	0.920, 0.04	0.914, 0.04
M3 (*bs*)	82.5, 8.8	0.847, 0.08	0.818, 0.10	0.814, 0.10
M1 (*pe*)	**92.5, 5.5**	**0.930, 0.05**	**0.927, 0.05**	**0.922, 0.05**
M2 (*pe*)	90.1, 6.2	0.909, 0.05	0.879, 0.05	0.894, 0.06
M3 (*pe*)	79.3, 7.8	0.807, 0.09	0.781, 0.09	0.775, 0.09
M1 (*lwpe*)	91.3, 7.5	0.915, 0.06	0.907, 0.08	0.906, 0.07
M2 (*lwpe*)	**91.4, 5.5**	**0.919, 0.05**	**0.908, 0.05**	**0.905, 0.06**
M3 (*lwpe*)	81.7, 7.2	0.842, 0.07	0.815, 0.08	0.811, 0.07
M1 (*lw*)	**92.9, 5.8**	**0.951, 0.03**	**0.921, 0.05**	**0.924, 0.05**
M2 (*lw*)	71.3, 6.0	0.773, 0.07	0.694, 0.08	0.738, 0.04
M3 (*lw*)	82.1, 8.5	0.074, 0.08	0.815, 0.09	0.813, 0.09

**Table 10:** Class prediction results using **third-person** environment and **K3HI Interaction Dataset**. (*bs*), (*pe*), (*lwpe*), and (*lw*) are different action selection methods (ref. Section 4, action selection); classification accuracy is abbreviated as Acc.

Model	Acc.	Recall	Precision	F1 Score
M1 (*bs*)	**83.0, 6.6**	**0.827, 0.07**	**0.816, 0.08**	**0.813, 0.08**
M2 (*bs*)	81.1, 3.3	0.796, 0.03	0.783, 0.03	0.780, 0.03
M3 (*bs*)	80.1, 3.1	0.796, 0.03	0.783, 0.02	0.777, 0.03
M1 (*pe*)	**82.7, 7.3**	**0.816, 0.08**	**0.815, 0.08**	**0.810, 0.08**
M2 (*pe*)	82.4, 3.9	0.825, 0.04	0.804, 0.04	0.805, 0.05
M3 (*pe*)	75.0, 5.7	0.762, 0.04	0.741, 0.05	0.738, 0.05
M1 (*lwpe*)	**82.1, 4.5**	**0.809, 0.04**	**0.800, 0.06**	**0.796, 0.05**
M2 (*lwpe*)	80.5, 7.8	0.794, 0.08	0.790, 0.10	0.784, 0.09
M3 (*lwpe*)	72.7, 8.3	0.731, 0.07	0.720, 0.07	0.712, 0.07
M1 (*lw*)	**80.8, 6.3**	**0.793, 0.07**	**0.775, 0.08**	**0.777, 0.08**
M2 (*lw*)	78.3, 6.3	0.803, 0.07	0.766, 0.07	0.764, 0.08
M3 (*lw*)	75.0, 7.1	0.758, 0.05	0.741, 0.06	0.736, 0.06

**Table 11:** Percentage of salient joints (mean, std dev) sampled by different variants of our models from the ground truth using **first-person** environment shown for (*pe*); (*bs*), (*lwpe*), and (*lw*) do not have sparsity. Interactions of shake hands and exchange object are abbreviated as Sh. Hands and Exc. obj., respectively.

Dataset	Model	Approach	Depart	Kick	Push	Sh. Hands	Exc. Obj.	Punch	Hug	Avg.
SBU	M1 [11]	48.9, 4.2	48.7, 3.9	46.6, 2.8	49.3, 2.1	49.8, 2.3	48.9, 2.2	49.9, 3.2	48.3, 3.0	48.8, 1.0
	M2	48.3, 3.5	48.4, 4.3	46.7, 3.3	49.2, 2.4	49.8, 2.7	48.4, 2.5	49.3, 2.5	47.4, 2.6	48.4, 1.0
	M3	48.5, 3.7	47.8, 4.4	46.3, 2.6	49.2, 2.2	48.7, 2.4	48.0, 1.9	49.3, 3.4	48.4, 2.3	48.3, 1.0
		Approach	Depart	Exc. obj.	Kick	Point	Punch	Push	Sh. Hands	Avg.
K3HI	M1 [11]	47.9, 3.0	47.6, 2.4	47.8, 3.0	45.8, 2.6	46.8, 4.4	47.4, 2.4	47.6, 2.0	46.3, 2.9	47.2, 1.0
	M2	48.4, 2.4	48.4, 2.1	48.3, 3.9	44.5, 2.5	44.8, 4.1	47.3, 2.7	47.9, 3.2	47.7, 4.2	47.1, 1.6
	M3	48.0, 2.2	47.9, 2.2	48.2, 3.2	44.6, 2.6	45.9, 4.4	47.5, 2.7	48.0, 3.3	47.0, 3.9	47.1, 1.3

**Table 12:** Percentage of salient joints (mean, std dev) sampled by different variants of our models from the ground truth using **third-person** environment shown for (*pe*); (*bs*), (*lwpe*), and (*lw*) do not have sparsity. Interactions of shake hands and exchange object are abbreviated as sh. hands and exc. obj., respectively.

Dataset	Model	Approach	Depart	Kick	Push	Sh. Hands	Exc. Obj.	Punch	Hug	Avg.
SBU	M1 [11]	47.5, 3.8	45.8, 4.6	45.1, 3.2	48.4, 2.7	47.7, 3.2	47.6, 2.8	48.7, 3.8	47.4, 2.9	47.3, 1.2
	M2	47.8, 4.5	45.6, 4.4	44.4, 3.0	48.6, 3.6	47.7, 3.8	47.5, 3.2	48.0, 3.9	47.1, 3.4	47.1, 1.4
	M3	46.7, 3.4	46.2, 4.4	44.6, 3.0	48.9, 3.1	47.9, 4.0	47.4, 2.5	47.7, 5.3	47.8, 3.4	47.1, 1.3
		Approach	Depart	Exc.	Kick	Point	Punch	Push	Sh. Hands	Avg.
K3HI	M1 [11]	47.2, 2.9	47.9, 3.0	46.9, 2.9	41.1, 3.5	39.9, 7.2	45.5, 3.1	45.8, 3.7	46.8, 5.5	45.1, 3.0
	M2	48.0, 3.6	48.6, 2.7	47.1, 2.6	41.0, 3.1	37.7, 6.4	44.6, 3.8	45.5, 3.1	45.9, 4.3	44.8, 3.7
	M3	47.2, 4.3	47.1, 3.0	45.9, 3.4	41.2, 3.7	40.4, 6.9	45.0, 2.5	44.3, 3.4	45.4, 4.4	44.6, 2.5

**Table 13:** Training time required (hours, iterations).

Model	SBU		K3HI	
	First Person	Third Person	First Person	Third Person
M1 (*bs*)	1.0, 7368	0.4, 4364	1.6, 5388	0.7, 2452
M2 (*bs*)	1.5, 9201	0.9, 8720	2.2, 5862	4.4, 17,499
M3 (*bs*)	0.4, 8250	0.3, 8018	0.7, 3459	0.5, 3310
M1 (*pe*)	1.8, 7146	0.5, 4166	5.2, 9154	1.8, 7199
M2 (*pe*)	2.7, 10,627	1.0, 8282	3.8, 6673	5.6, 17,430
M3 (*pe*)	0.6, 8207	0.3, 8105	1.0, 3255	0.4, 2926
M1 (*lwpe*)	1.2, 5512	0.5, 2844	2.7, 5421	2.5, 5832
M2 (*lwpe*)	3.4, 12,169	2.6, 13,030	6.5, 10,350	8.7, 17,499
M3 (*lwpe*)	0.5, 7727	0.4, 7586	0.8, 2887	0.6, 2685
M1 (*lw*)	1.4, 5203	1.3, 6889	4.6, 10,519	2.0, 3350
M2 (*lw*)	4.0, 17,999	3.4, 17,999	7.0, 12,352	8.2, 17,499
M3 (*lw*)	0.6, 8857	0.5, 8541	0.8, 2715	1.0, 3491

**Figure A2:**
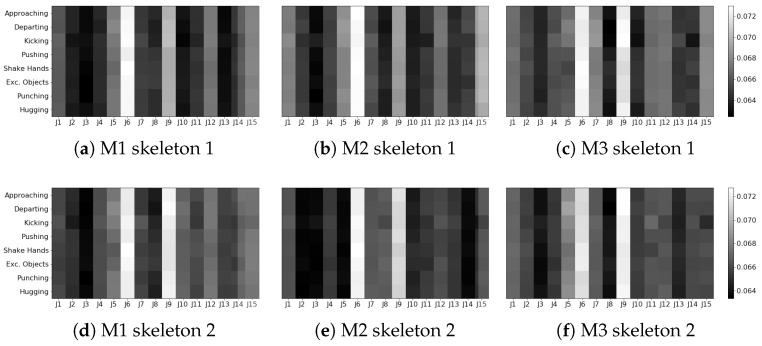
Salient joint distribution (dist.) over all interactions shown for skeleton 1 in (**a**–**c**) and the other skeleton in (**d**–**f**) for **first person, (*lwpe*)** environment using SBU Kinect interaction data.

**Figure A3:**
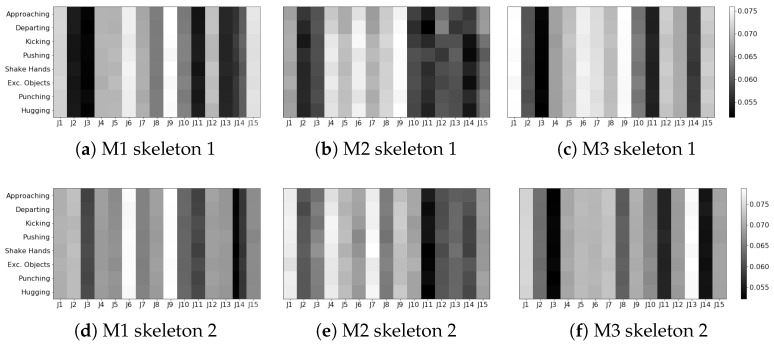
Salient joint distribution (dist.) over all interactions shown for skeleton 1 in (**a**–**c**) and the other skeleton in (**d**–**f**) for **first person, (*lw*)** environment using SBU Kinect interaction data.

**Figure A5:**
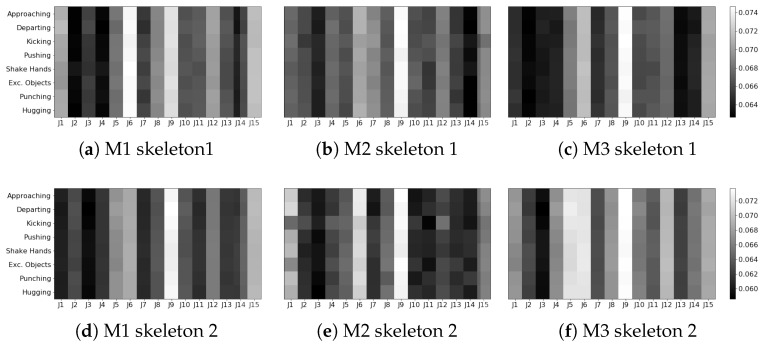
Salient joint distribution (dist.) over all interactions shown for skeleton 1 in (**a**–**c**) and the other skeleton in (**d**–**f**) for **third person, (*lwpe*)** environment using SBU Kinect interaction data.

**Figure A6:**
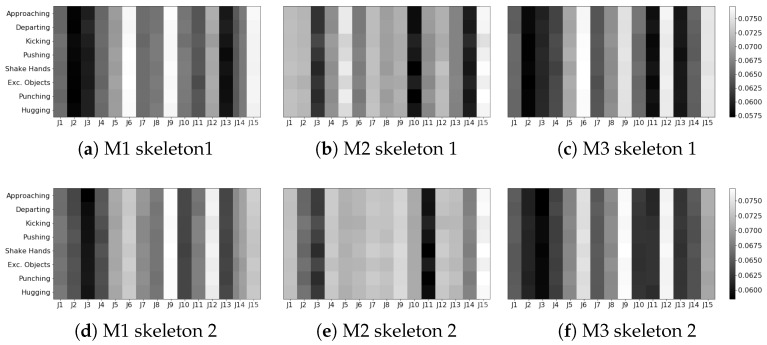
Salient joint distribution (dist.) over all interactions shown for skeleton 1 in (**a**–**c**) and the other skeleton in (**d**–**f**) for **third person, (*lw*)** environment using SBU Kinect interaction data.

**Figure A8:**
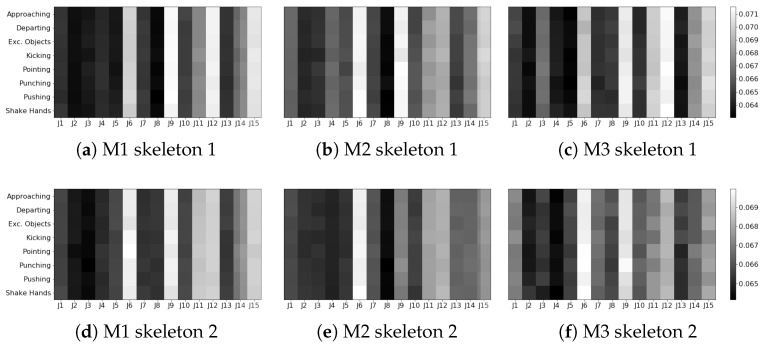
Salient joint distribution (dist.) over all interactions shown for skeleton 1 in (**a**–**c**) and the other skeleton in (**d**–**f**) for **first person, (*lwpe*)** environment using K3HI interaction data.

**Figure A9:**
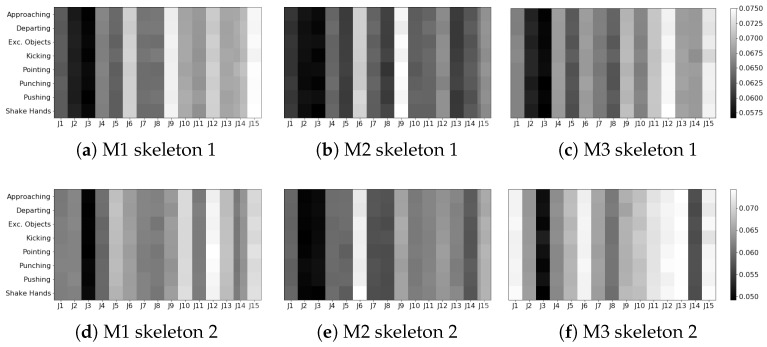
Salient joint distribution (dist.) over all interactions shown for skeleton 1 in (**a**–**c**) and the other skeleton in (**d**–**f**) for **first person, (*lw*)** environment using K3HI interaction data.

**Figure A11:**
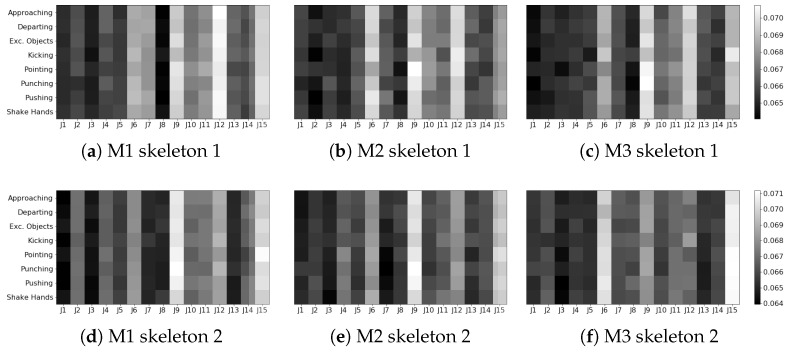
Salient joint distribution (dist.) over all interactions shown for skeleton 1 in (**a**–**c**) and the other skeleton in (**d**–**f**) for **third person, (*lwpe*)** environment using K3HI interaction data.

**Figure A12:**
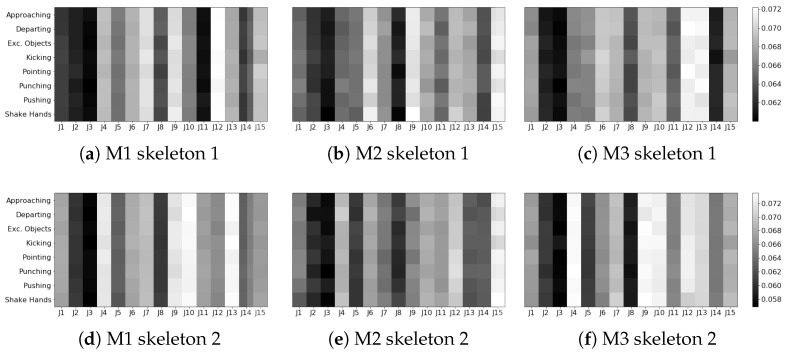
Salient joint distribution (dist.) over all interactions shown for skeleton 1 in (**a**–**c**) and the other skeleton in (**d**–**f**) for **third person, (*lw*)** environment using K3HI interaction data.

## Data Availability

The original contributions presented in the study are included in the article, further inquiries can be directed to the corresponding author.

## Appendix A. Loss Function Derivation

### Appendix A.1. Model M1

Following [11], we derive the objective function in Equation (9) from the objectives of multimodal VAE [51], variational RNN [50], and VAE for classification [49]. The generative and recognition models are factorized as

$$\begin{array}{c} p_{\theta }\left(X_{\leq T},y_{\leq T},z_{\leq T}|x_{\leq T}\right)\\ =\prod_{t=1}^{T}p_{\theta }\left(X_{t},y_{t}|z_{\leq t},x_{<t}\right)p_{\theta }\left(z_{t}|x_{<t},z_{<t}\right)q_{\phi }\left(z_{\leq T}|x_{\leq T}\right)\\ =\prod_{t=1}^{T}q_{\phi }\left(z_{t}|x_{\leq t},z_{<t}\right) \end{array}$$

The variational lower bound (ELBO) on the joint log-likelihood of the generated data, $\log p_{\theta }\left(X_{\leq T},y_{\leq T}|x_{\leq T}\right)$, is derived as

$$\begin{array}{c} E_{q_{\phi }\left(z_{\leq T}|x_{\leq T}\right)}\left[\log p_{\theta }\left(X_{\leq T},y_{\leq T}|x_{\leq T}\right)\frac{q_{\phi }\left(z_{\leq T}|x_{\leq T}\right)}{q_{\phi }\left(z_{\leq T}|x_{\leq T}\right)}\right]\\ =E_{q_{\phi }\left(z_{\leq T}|x_{\leq T}\right)}\left[\log\frac{p_{\theta }\left(X_{\leq T},y_{\leq T},z_{\leq T}|x_{\leq T}\right)}{p_{\theta }\left(z_{\leq T}|x_{\leq T}\right)}\frac{q_{\phi }\left(z_{\leq T}|x_{\leq T}\right)}{q_{\phi }\left(z_{\leq T}|x_{\leq T}\right)}\right]\\ =E_{q_{\phi }\left(z_{\leq T}|x_{\leq T}\right)}\left[\sum_{t=1}^{T}\log\frac{p_{\theta }\left(X_{t},y_{t}|z_{\leq t},x_{<t}\right)p_{\theta }\left(z_{t}|x_{<t},z_{<t}\right)}{p_{\theta }\left(z_{t}|x_{<t},z_{<t}\right)}\frac{q_{\phi }\left(z_{t}|x_{\leq t},z_{<t}\right)}{q_{\phi }\left(z_{t}|x_{\leq t},z_{<t}\right)}\right]\\ =E_{q_{\phi }\left(z_{\leq T}|x_{\leq T}\right)}\left[\sum_{t=1}^{T}\left[\log p_{\theta }\left(X_{t},y_{t}|z_{\leq t},x_{<t}\right)-\log\frac{q_{\phi }\left(z_{t}|x_{\leq t},z_{<t}\right)}{p_{\theta }\left(z_{t}|x_{<t},z_{<t}\right)}+\log\frac{q_{\phi }\left(z_{t}|x_{\leq t},z_{<t}\right)}{p_{\theta }\left(z_{t}|x_{<t},z_{<t}\right)}\right]\right]\\ \geq E_{q_{\phi }\left(z_{\leq T}|x_{\leq T}\right)}\left[\sum_{t=1}^{T}\log p_{\theta }\left(X_{t},y_{t}|z_{\leq t},x_{<t}\right)\right]-\sum_{t=1}^{T}D_{KL}\left(q_{\phi }\left(z_{t}|x_{\leq t},z_{<t}\right),p_{\theta }\left(z_{t}|x_{<t},z_{<t}\right)\right) \end{array}$$

We assume that the modalities are conditionally independent given the common latent variables [51] and all observations till the current time. Therefore,

$$\begin{array}{c} \log p_{\theta }\left(X_{t},y_{t}|z_{\leq t},x_{\leq t}\right)=\sum_{i=1}^{2}\log p_{\theta }\left(X_{t}^{\left(i\right)}|z_{\leq t},x_{<t}\right)+\log p_{\theta }\left(y_{t}|z_{\leq t},x_{<t}\right) \end{array}$$

Thus,

$$\begin{array}{c} \log p_{\theta }\left(X_{\leq T},y_{\leq T}|x_{\leq T}\right)\\ \geq E_{q_{\phi }\left(z_{\leq T}|x_{\leq T}\right)}\left[\sum_{t=1}^{T}\sum_{i=1}^{2}\log p_{\theta }\left(X_{t}^{\left(i\right)}|z_{\leq t},x_{<t}\right)+\log p_{\theta }\left(y_{t}|z_{\leq t},x_{<t}\right)\right]\\ \;\;\;\;-\sum_{t=1}^{T}D_{KL}\left(q_{\phi }\left(z_{t}|x_{\leq t},z_{<t}\right),p_{\theta }\left(z_{t}|x_{<t},z_{<t}\right)\right)\\ \geq E_{q_{\phi }\left(z_{\leq T}|x_{\leq T}\right)}\left[\sum_{t=1}^{T}\sum_{i=1}^{2}\lambda_{i}\log p_{\theta }\left(X_{t}^{\left(i\right)}|z_{\leq t},x_{<t}\right)+\lambda_{3}\log p_{\theta }\left(y_{t}|z_{\leq t},x_{<t}\right)\right]\\ \;\;\;\;-\beta \sum_{t=1}^{T}D_{KL}\left(q_{\phi }\left(z_{t}|x_{\leq t},z_{<t}\right),p_{\theta }\left(z_{t}|x_{<t},z_{<t}\right)\right) \end{array}$$

where $\lambda_{1}$, $\lambda_{2}$, $\lambda_{3}$, β are the weights balancing the terms. Assuming the class label does not change over time, we simplify the above expression as

$$\begin{array}{cc} E_{q_{\phi }\left(z_{\leq T}|x_{\leq T}\right)} & \left[\sum_{t=1}^{T}\sum_{i=1}^{2}\lambda_{i}\log p_{\theta }\left(X_{t}^{\left(i\right)}|z_{\leq t},x_{<t}\right)+\lambda_{3}\log p_{\theta }\left(y|z_{\leq T},x_{<T}\right)\right]\\ & -\beta \sum_{t=1}^{T}D_{KL}\left(q_{\phi }\left(z_{t}|x_{\leq t},z_{<t}\right),p_{\theta }\left(z_{t}|x_{<t},z_{<t}\right)\right) \end{array}$$

The pseudocodes are shown in Algorithms 1 and 2.

### Appendix A.2. Model M2

Here we derive the objective function in Equation (10). The generative and recognition models are factorized as

$$\begin{array}{c} p_{\theta }\left(X_{\leq T},y_{\leq T},z_{\leq T}|x_{\leq T}\right)\\ =\prod_{t=1}^{T}p_{\theta }\left(X_{t},y_{t}|z_{\leq t},x_{<t}\right)p_{\theta }\left(z_{t}|x_{<t},z_{<t}\right)q_{\phi }\left(z_{\leq T}|x_{\leq T},y_{\leq T}\right)\\ =\prod_{t=1}^{T}q_{\phi }\left(z_{t}|x_{\leq t},z_{<t},y_{t}\right) \end{array}$$

The variational lower bound (ELBO) on the joint log-likelihood of the generated data, $\log p_{\theta }\left(X_{\leq T},y_{\leq T}|x_{\leq T}\right)$, when the true label is given is derived as

$$\begin{array}{c} E_{q_{\phi }\left(z_{\leq T}|x_{\leq T},y_{\leq T}\right)}\left[\log p_{\theta }\left(X_{\leq T},y_{\leq T}|x_{\leq T}\right)\frac{q_{\phi }\left(z_{\leq T}|x_{\leq T},y_{\leq T}\right)}{q_{\phi }\left(z_{\leq T}|x_{\leq T},y_{\leq T}\right)}\right]\\ =E_{q_{\phi }\left(z_{\leq T}|x_{\leq T},y_{\leq T}\right)}\left[\log\frac{p_{\theta }\left(X_{\leq T},z_{\leq T},y_{\leq T}|x_{\leq T}\right)}{p_{\theta }\left(z_{\leq T}|x_{\leq T},y_{\leq T}\right)}\frac{q_{\phi }\left(z_{\leq T}|x_{\leq T},y_{\leq T}\right)}{q_{\phi }\left(z_{\leq T}|x_{\leq T},y_{\leq T}\right)}\right]\\ =E_{q_{\phi }\left(z_{\leq T}|x_{\leq T},y_{\leq T}\right)}\left[\sum_{t=1}^{T}\log\frac{p_{\theta }\left(X_{t}|z_{\leq t},x_{<t}\right)p_{\theta }\left(z_{t}|x_{<t},z_{<t},y_{t}\right)p_{\theta }\left(y_{t}\right)}{p_{\theta }\left(z_{t}|x_{<t},z_{<t},y_{t}\right)}\frac{q_{\phi }\left(z_{t}|x_{\leq t},z_{<t},y_{t}\right)}{q_{\phi }\left(z_{t}|x_{\leq t},z_{<t},y_{t}\right)}\right]\\ =E_{q_{\phi }\left(z_{\leq T}|x_{\leq T},y_{\leq T}\right)}[\sum_{t=1}^{T}[\log p_{\theta }\left(X_{t}|z_{\leq t},x_{<t}\right)+\log p_{\theta }\left(y_{t}\right)\\ \;\;\;\;-\log\frac{q_{\phi }\left(z_{t}|x_{\leq t},z_{<t},y_{t}\right)}{p_{\theta }\left(z_{t}|x_{<t},z_{<t},y_{t}\right)}+\log\frac{q_{\phi }\left(z_{t}|x_{\leq t},z_{<t},y_{t}\right)}{p_{\theta }\left(z_{t}|x_{<t},z_{<t},y_{t}\right)}]]\\ \geq E_{q_{\phi }\left(z_{\leq T}|x_{\leq T},y_{\leq T}\right)}\left[\sum_{t=1}^{T}\log p_{\theta }\left(X_{t}|z_{\leq t},x_{<t}\right)+\log p_{\theta }\left(y_{t}\right)\right]\\ \;\;\;\;-\sum_{t=1}^{T}D_{KL}\left(q_{\phi }\left(z_{t}|x_{\leq t},z_{<t},y_{t}\right),p_{\theta }\left(z_{t}|x_{<t},z_{<t},y_{t}\right)\right)\\ \geq E_{q_{\phi }\left(z_{\leq T}|x_{\leq T},y_{\leq T}\right)}\left[\sum_{t=1}^{T}\sum_{i=1}^{2}\log p_{\theta }\left(X_{t}^{\left(i\right)}|z_{\leq t},x_{<t}\right)+\log p_{\theta }\left(y_{t}\right)\right]\\ \;\;\;\;-\sum_{t=1}^{T}D_{KL}\left(q_{\phi }\left(z_{t}|x_{\leq t},z_{<t},y_{t}\right),p_{\theta }\left(z_{t}|x_{<t},z_{<t},y_{t}\right)\right) \end{array}$$

After adding the classification loss, the final objective function can be written as

$$\begin{array}{c} E_{q_{\phi }\left(z_{\leq T}|x_{\leq T},y_{\leq T}\right)}\left[\sum_{t=1}^{T}\sum_{i=1}^{2}\lambda_{i}\log p_{\theta }\left(X_{t}^{\left(i\right)}|z_{\leq t},x_{<t}\right)\right]+\log p_{\theta }\left(y_{t}\right)\\ \;\;\;\;\;\;\;\;\;\;\;\;\;\;\;\;\;\;\;\;-\beta \sum_{t=1}^{T}D_{KL}\left(q_{\phi }\left(z_{t}|x_{\leq t},z_{<t},y_{t}\right),p_{\theta }\left(z_{t}|x_{<t},z_{<t},y_{t}\right)\right)+\alpha \sum_{t=1}^{T}\log q_{\phi }\left(y_{t}|x_{\leq t}\right) \end{array}$$

where α controls the relative weight between generative and purely discriminative learning. Assuming the class label does not change over time, we simplify the above expression as

$$\begin{array}{c} E_{q_{\phi }\left(z_{\leq T}|x_{\leq T},y_{\leq T}\right)}\left[\sum_{t=1}^{T}\sum_{i=1}^{2}\lambda_{i}\log p_{\theta }\left(X_{t}^{\left(i\right)}|z_{\leq t},x_{<t}\right)\right]+\log p_{\theta }\left(y\right)\\ \;\;\;\;\;\;\;\;\;\;\;\;\;\;\;\;\;\;\;\;-\beta \sum_{t=1}^{T}D_{KL}\left(q_{\phi }\left(z_{t}|x_{\leq t},z_{<t},y_{t}\right),p_{\theta }\left(z_{t}|x_{<t},z_{<t},y_{t}\right)\right)+\alpha \log q_{\phi }\left(y|x_{\leq T}\right) \end{array}$$

The pseudocode is shown in Algorithm 3.

### Appendix A.3. Model M3

Here we derive the objective function in Equation (11). The generative and recognition models are factorized as

$$\begin{array}{c} p_{\theta }\left(X_{\leq T},z_{\leq T}|x_{\leq T}\right)=\prod_{t=1}^{T}p_{\theta }\left(X_{t}|z_{\leq t},x_{<t}\right)p_{\theta }\left(z_{t}|x_{<t},z_{<t}\right)\\ \;q_{\phi }\left(z_{\leq T}|x_{\leq T}\right)=\prod_{t=1}^{T}q_{\phi }\left(z_{t}|x_{\leq t},z_{<t}\right) \end{array}$$

The variational lower bound (ELBO) on the log-likelihood of the generated data, $\log p_{\theta }\left(X_{\leq T}|x_{\leq T}\right)$, is derived as

$$\begin{array}{c} E_{q_{\phi }\left(z_{\leq T}|x_{\leq T}\right)}\left[\log p_{\theta }\left(X_{\leq T}|x_{\leq T}\right)\frac{q_{\phi }\left(z_{\leq T}|x_{\leq T}\right)}{q_{\phi }\left(z_{\leq T}|x_{\leq T}\right)}\right]\\ =E_{q_{\phi }\left(z_{\leq T}|x_{\leq T}\right)}\left[\log\frac{p_{\theta }\left(X_{\leq T},z_{\leq T}|x_{\leq T}\right)}{p_{\theta }\left(z_{\leq T}|x_{\leq T}\right)}\frac{q_{\phi }\left(z_{\leq T}|x_{\leq T}\right)}{q_{\phi }\left(z_{\leq T}|x_{\leq T}\right)}\right]\\ =E_{q_{\phi }\left(z_{\leq T}|x_{\leq T}\right)}\left[\sum_{t=1}^{T}\log\frac{p_{\theta }\left(X_{t}|z_{\leq t},x_{<t}\right)p_{\theta }\left(z_{t}|x_{<t},z_{<t}\right)}{p_{\theta }\left(z_{t}|x_{<t},z_{<t}\right)}\frac{q_{\phi }\left(z_{t}|x_{\leq t},z_{<t}\right)}{q_{\phi }\left(z_{t}|x_{\leq t},z_{<t}\right)}\right]\\ =E_{q_{\phi }\left(z_{\leq T}|x_{\leq T}\right)}\left[\sum_{t=1}^{T}\left(\log p_{\theta }\left(X_{t}|z_{\leq t},x_{<t}\right)-\log\frac{q_{\phi }\left(z_{t}|x_{\leq t},z_{<t}\right)}{p_{\theta }\left(z_{t}|x_{<t},z_{<t}\right)}+\log\frac{q_{\phi }\left(z_{t}|x_{\leq t},z_{<t}\right)}{p_{\theta }\left(z_{t}|x_{<t},z_{<t}\right)}\right)\right]\\ \geq E_{q_{\phi }\left(z_{\leq T}|x_{\leq T}\right)}\left[\sum_{t=1}^{T}\log p_{\theta }\left(X_{t}|z_{\leq t},x_{<t}\right)\right]-\sum_{t=1}^{T}D_{KL}\left(q_{\phi }\left(z_{t}|x_{\leq t},z_{<t}\right),p_{\theta }\left(z_{t}|x_{<t},z_{<t}\right)\right)\\ \geq E_{q_{\phi }\left(z_{\leq T}|x_{\leq T}\right)}\left[\sum_{t=1}^{T}\sum_{i=1}^{2}\log p_{\theta }\left(X_{t}^{\left(i\right)}|z_{\leq t},x_{<t}\right)\right]-\sum_{t=1}^{T}D_{KL}\left(q_{\phi }\left(z_{t}|x_{\leq t},z_{<t}\right),p_{\theta }\left(z_{t}|x_{<t},z_{<t}\right)\right)\\ \geq E_{q_{\phi }\left(z_{\leq T}|x_{\leq T}\right)}\left[\sum_{t=1}^{T}\sum_{i=1}^{2}\lambda_{i}\log p_{\theta }\left(X_{t}^{\left(i\right)}|z_{\leq t},x_{<t}\right)\right]-\beta \sum_{t=1}^{T}D_{KL}\left(q_{\phi }\left(z_{t}|x_{\leq t},z_{<t}\right),p_{\theta }\left(z_{t}|x_{<t},z_{<t}\right)\right) \end{array}$$

where $\lambda_{1}$, $\lambda_{2}$, β are the weights balancing the terms.

After adding the classification loss, the final objective function can be written as

$$\begin{array}{cc} E_{q_{\phi }\left(z_{\leq T}|x_{\leq T}\right)} & \left[\sum_{t=1}^{T}\sum_{i=1}^{2}\lambda_{i}\log p_{\theta }\left(X_{t}^{\left(i\right)}|z_{\leq t},x_{<t}\right)\right]\\ & -\beta \sum_{t=1}^{T}D_{KL}\left(q_{\phi }\left(z_{t}|x_{\leq t}\right),p_{\theta }\left(z_{t}\right)\right)+\log q_{\pi }\left(y|X_{1:T}\right) \end{array}$$

where $q_{\pi }\left(y|X_{1:T}\right)$ is the classification model.

## Appendix B. Experimental Results (Details)

Details of our experimental results from the efficiency evaluation are provided here.

### Appendix B.1. Efficiency evaluation

Percentage of salient joints sampled by each of our model variants from the ground truth are shown in Table 11 and Table 12. The distribution of salient regions for all interaction classes for each skeleton are shown in Figure A1, Figure A2, Figure A3, Figure A4, Figure A5, Figure A6, Figure A7, Figure A8, Figure A9, Figure A10, Figure A11 and Figure A12. In both sets of tables and figures, results from first-person and third-person environments for the SBU Kinect and K3HI datasets are shown. The figures show a sparse saliency distribution in most cases.
